# Synthesis of 3-(arylamino) quinazoline-2,4(1*H*,3*H*)-dione derivatives *via* TBHP/I_2_: Molecular docking, MD simulation, DFT, and pharmacological evaluation as MTH1 inhibitors

**DOI:** 10.1371/journal.pone.0335707

**Published:** 2025-11-13

**Authors:** Moeid Goudarzi Karim, Morteza Mehrdad, Davood Gheidari, Nazanin Zahra Gheidari

**Affiliations:** Department of Chemistry, Faculty of Science, University of Guilan, Rasht, Iran; Al-Azhar University Faculty of Pharmacy for Boys, EGYPT

## Abstract

A series of novel derivatives of 3-(arylamino) quinazoline-2,4(1*H*,3*H*)-dione were synthesized with moderate to good yields (20%−70%) using *t*-butyl hydroperoxide (TBHP) and iodine. Their efficacy against MutT homologue 1 (MTH1) was evaluated using in *silico* methods. Density functional theory (DFT) analysis, utilizing the B3LYP/6-311G (2df, p) basis set, indicated a promising reactivity profile for the synthesized compounds. The highest occupied molecular orbital (HOMO) regions associated with the phenylhydrazine group serve as sites for electron donation, functioning as electron-rich nucleophiles. Docking analysis with MTH1 enzymes revealed that all compounds exhibited docking scores ranging from −5.77 to −7.24, indicating favorable binding affinities. Among these, compound **(3d)**, with an energy of −7.24 kcal/mol, demonstrated the strongest binding affinity. Importantly, the Generalized Born and Surface Area Solvation (MM-GBSA) rescoring aligned with the docking data, reinforcing the reliability of the predicted binding modes and highlighting these compounds as promising MTH1 inhibitors. Molecular dynamics (MD) simulations indicated that Tyr7, Thr8, Lys23, and Trp117 exhibited a notably high interaction fraction, suggesting that these residues might be critical for the binding affinity of compound **(3d)**. The analysis of absorption, distribution, metabolism, excretion, and toxicity (ADMET) properties indicated that all compounds possess a favorable pharmacological profile and comply with Lipinski’s Rule of Five (Ro5), as well as the Ghose, Veber, and Egan rules. Additionally, they are capable of human intestinal absorption (HIA) and exhibit no liver toxicity, whereas BAY-707 is anticipated to exhibit hepatotoxicity.

## Introduction

**Fig 1:**
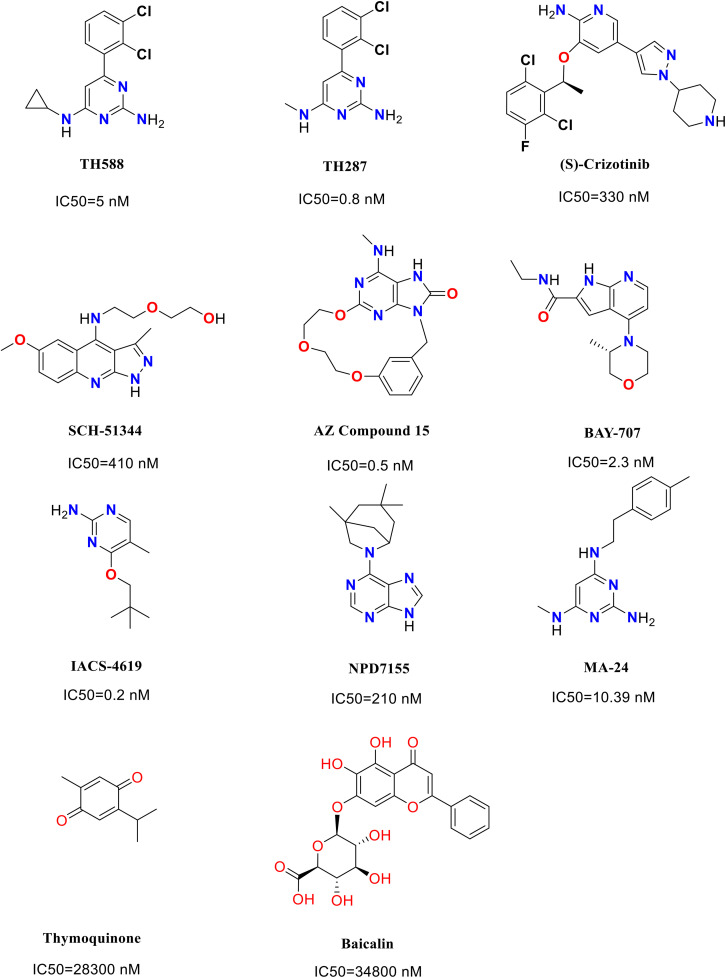
Structure of MTH1 inhibitors.

Breast cancer represents a major public health concern among women, being the second leading cause of cancer-related mortality in the United States [1]. It is commonly diagnosed through clinical indicators such as palpable masses within breast tissue, morphological alterations in breast architecture, skin dimpling, and fluid discharge from the nipple. While conventional chemotherapeutic agents demonstrate significant inhibitory effects on breast cancer, they also inflict collateral damage on healthy cells, which can increase the risk of adverse effects [2]. Furthermore, the emergence of drug resistance complicates treatment efficacy, diminishing the therapeutic potential of these agents [3]. Although targeted therapies have shown considerable success in specific subsets of breast cancer patients, their limited specificity restricts broader applicability across the entire patient population [4]. To address these therapeutic limitations, targeting the frequently observed aberrant characteristics of cancer cells presents a viable strategy that could lead to more comprehensive cancer treatment. Reactive oxygen species (ROS), produced as byproducts of oxidative phosphorylation, have dual effects on cancer cells. Excess ROS accumulation can result from abnormal cellular proliferation, leading to the oxidation of deoxynucleoside triphosphates (dNTPs) and the formation of oxidized derivatives such as 8-oxo-dGTP and 2-OH-dATP [5]. In this context, MTH1 has emerged as a promising target in cancer therapy. This enzyme sanitizes oxidized dNTP pools, converting 8-oxo-dGTP to 8-oxo-dGMP to prevent the incorporation of damaged bases during DNA replication [6]. Investigating the molecular mechanisms of MTH1 reveals that within its active site, 8-oxo-dGMP adopts an anti-conformation, establishing π-stacking interactions with Trp117 and Phe72. The Watson-Crick face of 8-oxo-dGMP is specifically recognized by Asp119 and Asp120 through hydrogen bonds to the 6-O, 1-NH, and 2-NH_2_ functional groups [7]. Additionally, Asn33, located at the base of the purine ring of 8-oxo-dGMP, forms two hydrogen bonds with the 2-NH_2_ and 3-N groups. Notably, the Trp117Ala mutation significantly impairs the hydrolytic activity of MTH1 on both 8-oxo-dGTP and 2-OH-dATP, underscoring the vital role of Trp117 in the enzyme’s function [8]. Moreover, Asn33 is also critical for MTH1 activity; the Asn33Glu mutation completely abolishes hydrolysis of 8-oxo-dGTP, while the Asn33Ala mutation reduces enzymatic activity to only 14% of the wild-type level [9]. Collectively, Asp119, Asp120, Asn33, and Trp117 are essential for substrate recognition and excision by MTH1, representing key residues for the pharmacological inhibition of this enzyme. Overexpression of MTH1 has been documented in breast cancer and is particularly pronounced in certain subtypes of this disease [10–11]. The variability in MTH1 expression is closely associated with ROS levels within cancer cells, where cells exhibiting elevated ROS concentrations increasingly depend on MTH1 for survival due to heightened oxidative stress [12]. Consequently, targeting MTH1 to enhance the accumulation of oxidized nucleotides within cells, thereby inducing DNA damage, can be regarded as an innovative strategy for the eradication of breast cancer. In recent decades, numerous MTH1 inhibitors have undergone clinical evaluation, yielding promising results regarding their anticancer efficacy. Among these, the initial small molecule inhibitors specifically designed to target MTH1, such as TH287 and TH588, have attracted significant attention due to their potent anticancer properties [2]. This growing interest among researchers in the field of MTH1 inhibition is supported by prior studies demonstrating that TH588 effectively inhibits the growth of malignant melanomas in murine models, as well as various other cancers, including SW480 colorectal tumors, glioblastomas, and MCF7 breast cancer tumors [13]. A detailed examination of the molecular mechanisms reveals that the aminopyrimidine moiety in TH287 and TH588 forms crucial hydrogen bonding interactions within the active site with key residues such as Asn 33, Asp 119, and Asp 120. Additionally, the aminomethyl substituent in TH287, along with the corresponding amino group in TH588, plays a critical role in optimizing both binding affinity and substrate specificity. Notably, the hydrogen bond established between the primary amine and Asp 119 is believed to structurally and functionally mimic the 6-enol tautomer of 8-oxodGTP, a molecule previously suggested to be pivotal for specificity within this enzymatic context [7,14]. In 2014, Crizotinib, a member of a kinase inhibitor collection, was identified as an effective dual inhibitor of *c*-MET/ALK kinases [15]. Notably, the S-enantiomer of Crizotinib not only exhibits inhibitory effects on MTH1 but also demonstrates significant potential in reducing tumor size in carcinoma xenograft models and in suppressing osteosarcoma growth [16]. In 2016, a Toll-like receptor 7 (TLR7) agonist with anticancer potential was found to interact with MTH1, leading to the design and synthesis of a series of compounds based on the structural framework of the TLR7 agonist protein. Among these, three compounds displayed binding affinity for MTH1, and five additional MTH1 compound complex structures were successfully elucidated [17]. In 2018, Rahm et al. reported a novel and potent MTH1 inhibitor, BAY-707 [18]. In 2019, 3-isomangostin was identified in a study of compound cocktails, with its binding mode to MTH1 confirmed through X-ray crystallography [19] Taiyab *et al*. [20] have shown that thymoquinone (TQ) and baicalin (BC) function as inhibitors of the MTH1 enzyme. Their research indicates that both TQ and BC bind effectively to the active site of MTH1, leading to the formation of stable complexes. The administration of TQ and BC in breast cancer cells resulted in a significant reduction in cellular growth and proliferation, with IC_50_ values determined to be 28.3 µM and 34.8 µM, respectively, and triggered apoptotic pathways. Moreover, TQ and BC were found to increase the production of reactive ROS in MCF7 cells, inducing considerable oxidative stress that ultimately culminated in cell death (Fig 1).

**Fig 2:**
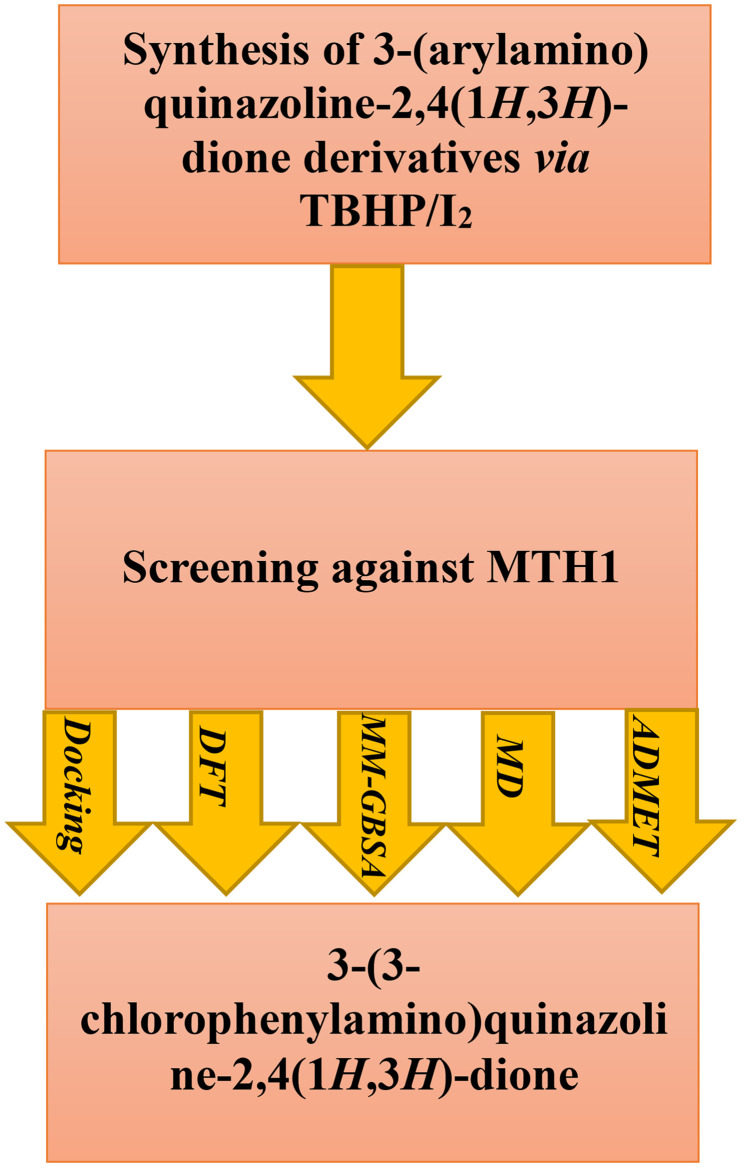
Workflow for 3‑(arylamino)quinazoline‑2,4(1*H*,3H)‑dione derivatives as prospective MTH1 inhibitors: synthesis, DFT, docking/MM‑GBSA, MD, and ADMET evaluation.

Consequently, there is a growing interest among scientists in the development and synthesis of MTH1 inhibitors. Drawing upon prior research focused on bioactive heterocyclic compounds [21], we undertook a study aimed at synthesizing derivatives of 3-(arylamino)quinazoline-2,4(1*H*,3*H*)-dione through the reaction of isatin and phenylhydrazine, informed by the pharmacophore features of MTH1 inhibitors. Acknowledging the importance of bioinformatics approaches such as molecular docking, MM-GBSA, and MD simulations, we employed these methods to analyze the target receptor interactions of the compounds. This approach helps to elucidate their structural similarities and binding characteristics, serving as a valuable tool for screening potential bioactive compounds and predicting their biological activities. Such computational techniques allow for detailed atomic-level interaction modeling, significantly accelerating the drug discovery process while reducing reliance on lengthy and resource-intensive experimental methods. Following this, drug-likeness and ADMET predictions were performed to identify compounds with desirable pharmacokinetic and safety profiles. Additionally, DFT calculations were conducted, which are fundamental in computational chemistry. DFT provides a quantum mechanical framework for accurately describing electronic structures, enabling precise prediction of molecular geometries, electronic properties, and reaction mechanisms. This method is particularly effective in evaluating the energetics of molecular interactions, crucial for understanding binding affinities and stability in drug design (Fig 2).

## Result and discussion

### Chemistry

According to the literature, compound **(3a)** [22] has previously been synthesized using another method. However, we successfully developed derivatives of 3-(arylamino) quinazoline-2,4(1*H*,3*H*)-dione **(3a)** through a simple and efficient synthetic approach, as illustrated in S2 File. Initially, isatin (**1a**, 1 mmol) and phenyl hydrazine (**2a**, 1 mmol) were combined in various solvents at different temperatures without the use of oxidants to determine the optimal reaction conditions. Our findings indicated that the reaction did not proceed in any solvent in the absence of an oxidant. Subsequently, as detailed in Table 1, we evaluated Cu(NO_3_)_2_ as the oxidant across different solvents and temperatures to identify the most suitable reaction conditions. Methanol (MeOH) was determined to be the optimal solvent, and the ideal reaction temperature was found to be the reflux temperature, resulting in the formation of compound **(3a)** with a yield of 15% (Entry 6, Table 1). Following this, the reaction was performed using MeOH in conjunction with various oxidants to further optimize the conditions. Ultimately, the most favorable conditions were achieved with the use of TBHP and iodine, leading to a yield of 70% (Entry 5, Table 2). Utilizing these optimized conditions, we successfully synthesized additional derivatives of 3-(arylamino) quinazoline-2,4(1*H*,3*H*)-dione **(3a–e)**.

**Table 1:** Reaction conditions optimized for (3a) ^a^.

Entry	Solvent	Temp (^°^C)	Time (h)	Oxidant	Yield (%)
**1**	H_2_O	r. t.	24	Cu(NO_3_)_2_	nr
**2**	H_2_O	reflux	24	Cu(NO_3_)_2_	nr
**3**	EtOH	r. t.	24	Cu(NO_3_)_2_	nr
**4**	EtOH	reflux	24	Cu(NO_3_)_2_	10
**5**	MeOH	r. t.	24	Cu(NO_3_)_2_	nr
**6**	**MeOH**	**reflux**	**24**	**Cu(NO**_**3**_)_**2**_	**15**
**7**	Acetone	reflux	24	Cu(NO_3_)_2_	nr

^*a*^ Reagents and conditions: **1a** (1 mmol), **2a** (1 mmol), oxidant (1 mmol), solvent (10 mL). nr = no reaction.

**Table 2:** Various oxidants for generation of compound (3a).

Entry	Oxidant	Solvent	Time (h)	Yield (%)
**1**	H_2_O_2_/NaOH	MeOH	24	nr
**2**	Cu(SO_4_)	MeOH	24	nr
**3**	Cu(OAc)_2_	MeOH	24	nr
**4**	Cu(NO_3_)_2_	MeOH	24	15
**5**	**I**_**2**_ **/ TBHP**	**MeOH**	**5**	**70**
**6**	I_2_/ TBHP	EtOH	7	60

**Fig 3:**
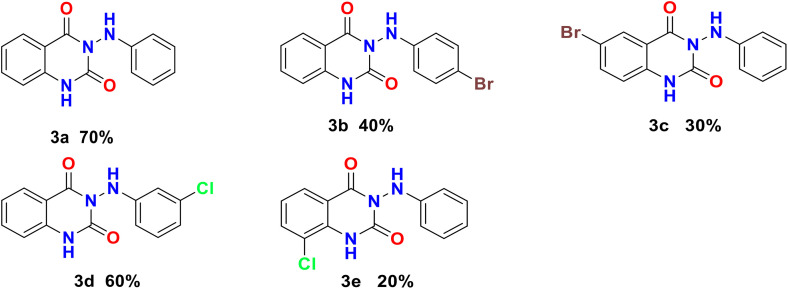
The detailed structures and percent yields of the synthesized derivatives (3a-e). A proposed mechanism for the synthesis of 3-(phenylamino) quinazoline-2,4(1*H*,3*H*)-dione **(3a)** is depicted in S3 File. Initially, the reaction mechanism for the synthesis of 3-(arylamino) quinazoline-2,4(1*H*,3*H*)-dione derivatives begins with the nucleophilic attack of the amino group of phenyl hydrazine **(2a)** on the carbonyl carbon of isatin **(1a)**, resulting in the formation of a hydrazone intermediate **(4a)** [23]. Subsequently, TBHP is introduced as an oxidizing agent, which interacts with the hydrazone **(4a)** to form a peroxy intermediate **(5a)**. This intermediate undergoes an iodination step with iodine, resulting in the formation of another reactive species while releasing hydrogen iodide as a byproduct. Finally, the reaction culminates in the elimination of HI from the intermediate, leading to the cyclization and formation of the target compound, 3-(phenylamino) quinazoline-2,4(1*H*,3*H*)-dione **(3a)**.

The detailed structures of the synthesized derivatives are depicted in Fig 3. The structural elucidation of 3-(arylamino) quinazoline-2,4(1*H*,3*H*)-dione derivatives **(3a–e)** was achieved through the analysis of spectroscopic data obtained from mass, IR spectroscopy, and both ^1^H and ^13^C nuclear NMR spectroscopy. For instance, the IR spectrum of the product 3-(phenylamino) quinazoline-2,4(1*H*,3*H*)-dione **(3a)** exhibited N-H stretching vibrations at 3442 and 3277 cm^−1^, carbonyl stretching vibrations associated with the amide group at 1739 cm^−1^, urea carbonyl stretching vibrations at 1668 cm^−1^, and C-N stretching vibrations of the amino group at 1276, 1239, and 1163 cm^−1^. The ^1^H-NMR spectrum of compound **(3a)** revealed a chemical shift (δ, ppm) for the H-1 proton at 11.65 ppm, appearing as a broad singlet, while the (NHPh) proton was observed at 8.49 ppm as a singlet. The H-5 proton appeared at 7.96 ppm as a doublet (*J* = 6.8 Hz), the H-7 proton at 7.71 ppm as a triplet (*J* = 7.2 Hz), and the H-6 protons at 7.25 ppm as triplets (*J* = 7.2 Hz). The H-8 proton was noted at 7.24 ppm as a doublet (*J* = 8.0 Hz), while the H-3’ and H-5’ protons were observed at 7.15 ppm as triplets (*J* = 7.8 Hz). The H-4’ proton appeared at 6.77 ppm as triplets (*J* = 7.4 Hz), and the H-2’ and H-6’ protons were detected at 6.65 ppm as a doublet (*J* = 8.0 Hz). The ^13^C NMR spectrum of compound **(3a)** displayed chemical shifts (δ, ppm) for the C-4 carbonyl amide carbon at 162.0 ppm, the C-2 urea carbonyl carbon at 150.4 ppm, C-1’ at 147.8 ppm, C-2’ and C-6’ at 129.3 ppm, C-4’ at 128.1 ppm, C-3’ and C-5’ at 112.8 ppm, C-8a at 139.8 ppm, C-7 at 135.8 ppm, C-5 at 123.2 ppm, C-6 at 119.8 ppm, C-8 at 115.9 ppm, and C-4a at 114.5 ppm. The mass spectrum of compound **(3a)** exhibited a molecular ion peak at m/z 253, which is consistent with the proposed structure. Additionally, a molecular ion peak at m/z 209 corresponds to a fragment resulting from the loss of a CO_2_ group.

### Computational study

**Fig 4:**
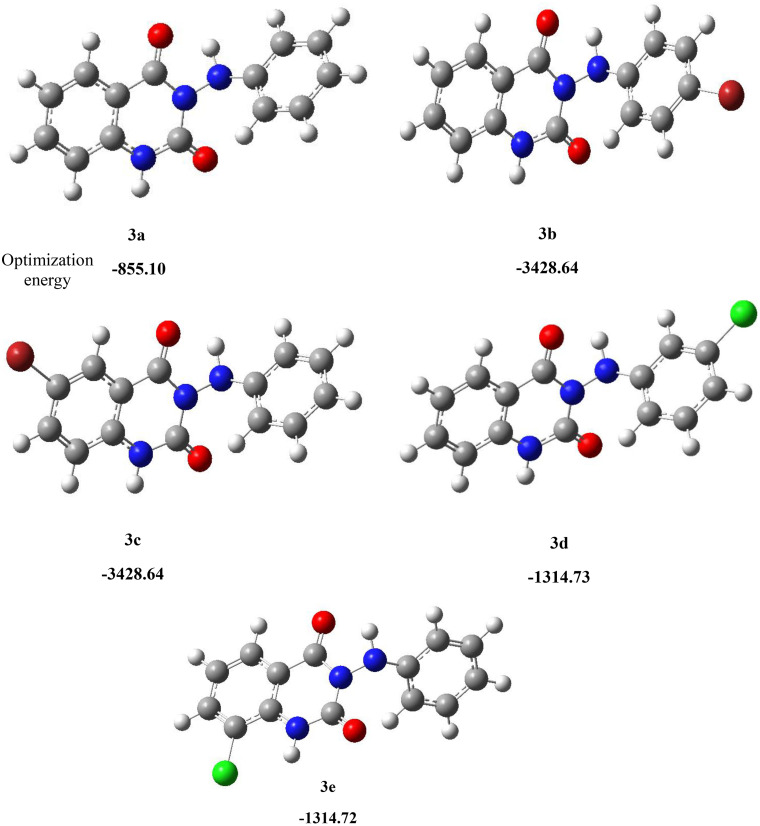
Optimized molecular structures of the 3-(arylamino) quinazoline-2,4(1*H*,3*H*)-diones derivatives (3a–e) using DFT/B3LYP/6-311G(2df,p).

**DFT.** DFT is a computational method in quantum chemistry used to investigate the electronic structure and properties of molecules and materials. This approach is based on electron density rather than the wave function, allowing for efficient calculations of molecular properties. DFT is widely employed in molecular simulations and structure optimization due to its balance of accuracy and computational efficiency. In this study, the B3LYP/6-311G (2df, p) basis set was utilized for the optimization of the molecular structures of 3-(arylamino) quinazoline-2,4(1*H*,3*H*)-dione derivatives **(3a–e)** using the Gaussian 09W program [24] and Gauss View 5.0. Molecular structure optimization is a process in which the geometry of a molecule is adjusted to minimize its total energy. This involves refining the positions of atoms and bond angles to achieve a stable and optimal structure. There were no instances of imaginary frequencies found, and the geometries of the selected compounds were adjusted to lower energy gradients, indicating that all compounds were indeed local minima. The optimized structures of the compounds are depicted in Fig 4, allowing for accurate predictions of their stable configurations and associated physical and chemical properties.

**Fig 5:**
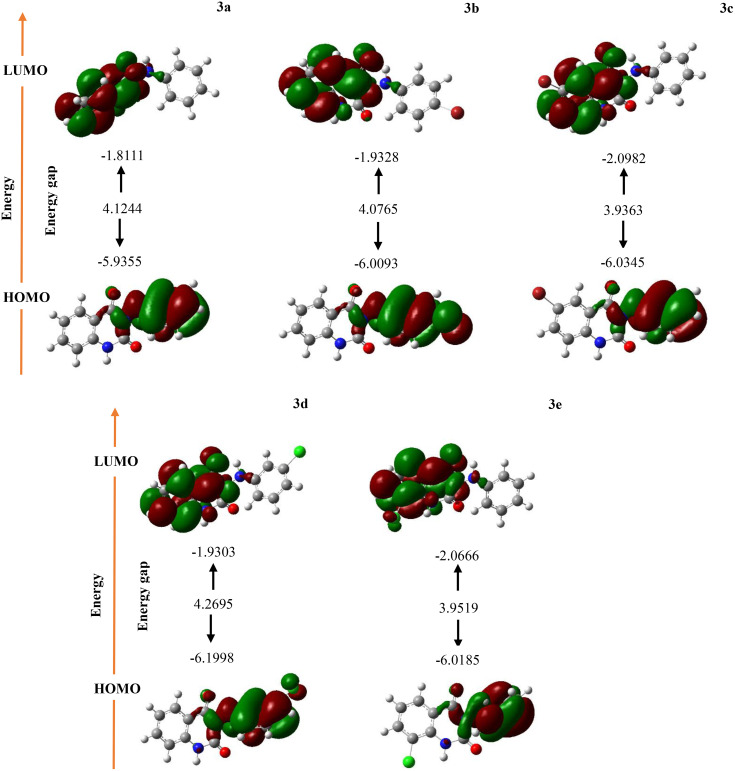
FMOs of the 3-(arylamino) quinazoline-2,4(1*H*,3*H*)-diones derivatives (3a-e).

Frontier molecular orbitals (FMOs) analysis provides valuable insights into the electronic characteristics, stability, and reactivity of molecular compounds [25]. Understanding these properties is vital for designing and developing new materials and pharmaceuticals, enabling researchers to predict compound behavior in various chemical environments. The key parameters in this analysis include the HOMO, LUMO, and the energy gap (ΔE). The HOMO values range from −6.1998 **(3d)** to −5.9355 **(3a)**, with compound **(3d)** showing the most favorable HOMO energy, indicating a higher tendency to donate electrons. In contrast, the LUMO values span from −1.8111 **(3a)** to −2.0982 **(3c)**, with **(3c)** having the most favorable position for accepting electrons. The energy gaps (∆E) range from 3.9363 **(3c)** to 4.2695 **(3d)**. A smaller energy gap in **(3c)** suggests that it is more reactive and facilitates for electronic transitions, likely leading to greater chemical reactivity. In the provided FMOs, distinct regions can be identified as electrophilic and nucleophilic. As illustrated in Fig 5, the HOMO regions associated with the phenylhydrazine group can serve as sites for electron donation, functioning as electron-rich nucleophiles. Conversely, the quinazoline-2,4(1*H*,3*H*)-dione moiety can act as an electrophilic site for electron acceptance. This distinction highlights the complementary roles of these molecular components in facilitating chemical reactivity.

ΔE in FMOs encompasses a spectrum of additional critical parameters that facilitate the analysis and prediction of the chemical and physical properties of molecules. One such descriptor is the ionization potential (*I*), which signifies the energy required to extract an electron from an atom or molecule in the gaseous state, providing insights into the atom’s proclivity to lose electrons. Complementing this is the electron affinity (*A*), which quantifies the energy liberated when an electron is appended to an atom, indicating the atom’s tendency to attract electrons. Another essential descriptor is electronegativity (*χ*), defined as the mean of the *I* and *A*, reflecting an atom’s capability to attract electrons in chemical bonds. Additionally, chemical hardness (*η*) quantifies a molecule’s resistance to electronic alterations, while chemical softness (*S*) serves as the inverse of hardness, indicating the molecule’s propensity to accept or donate electrons. The chemical potential (*μ*) denotes the change in energy associated with the system, and electrophilicity (*ω*) measures a molecule’s inclination to accept electrons and engage in electrophilic reactions. These descriptors empower researchers to anticipate the chemical behavior of molecules under various conditions. The energetic parameters of 3-(arylamino) quinazoline-2,4(1*H*,3*H*)-dione derivatives **(3a-e)** are elucidated in Table 3. The ionization potentials reveal a consistent trend, with values ranging from 5.9355 **(3a)** to 6.1998 **(3d)**. A higher ionization potential in **(3d)** suggests that it is more challenging to ionize relative to the others. The *A* values span from 1.8111 **(3a)** to 2.0982 **(3c)**, indicating that **(3c)** possesses a heightened tendency to capture electrons. *χ* values are notably similar, ranging from 3.8733 **(3a)** to 4.0664 **(3c)**, with compound **(3c)** exhibiting the highest *χ*, signifying a stronger attraction to electrons in bonds. The *η* demonstrates slight variability, with values ranging from 1.9682 **(3c)** to 2.1348 **(3d)**. The greater hardness of **(3d)** implies that it is more stable and less reactive. *S* is inversely correlated with hardness, with values extending from 0.2342 **(3d)** to 0.2540 **(3c)**, suggesting that **(3c)** is the softest and most reactive. The *μ* values are negative for all compounds, indicating that all are thermodynamically favorable for electron donation. *ω* ranges from 3.6375 **(3a)** to 4.2008 **(3c)**, indicating that compound **(3c)** is the most electrophilic, thus more likely to attract nucleophiles. The polarizability values furnish further insight into the responsiveness of these compounds in the presence of an electric field, with **(3c)** being the highest at 206.17, which suggests that it can become readily polarized and may exhibit enhanced reactivity. The dipole moments vary significantly, from 3.4161 **(3c)** to 6.6364 **(3d)**, indicating that compound **(3d)** displays the highest polarity, which could contribute to stronger intermolecular interactions and influence solubility and chemical behavior.

**Table 3:** Energetic parameters of the 3-(arylamino)quinazoline-2,4(1*H*,3*H*)-dione derivatives (3a-e).

	3e	3d	3c	3b	3a
***I* (eV)**	6.0185	6.1998	6.0345	6.0093	5.9355
***A* (eV)**	2.0666	1.9303	2.0982	1.9328	1.8111
**χ (eV)**	4.0426	4.0651	4.0664	3.9711	3.8733
**(eV) *η***	1.9760	2.1348	1.9682	2.0383	2.0622
***S* (eV**^**-1**^)	0.2530	0.2342	0.2540	0.2453	0.2424
***µ* (eV)**	−4.0426	−4.0651	−4.0664	−3.9711	−3.8733
***ω* (eV)**	4.1354	3.8705	4.2008	3.8684	3.6375
**Polarizability (α)**	195.61	197.07	206.17	205.37	181.33
**Dipole moment**	3.7767	6.6364	3.4161	6.2455	4.5384

### Molecular docking study

**Fig 6:**
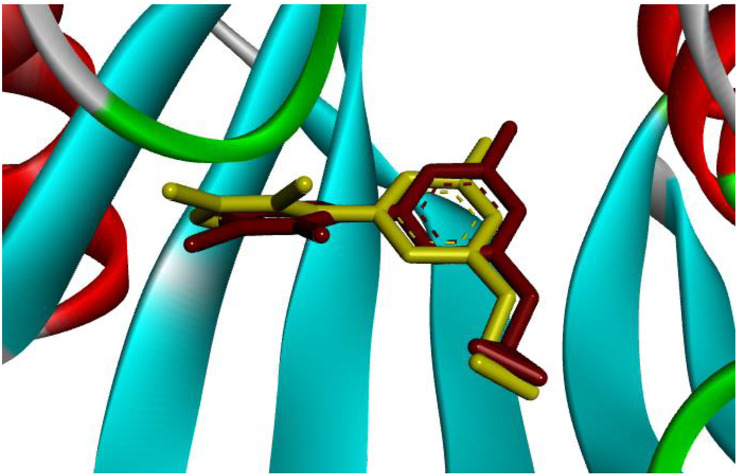
Superimposition of the native ligand (shown in yellow) and the re-docked ligand (colored red).

Molecular docking is a computational technique employed to predict interactions between molecules, particularly between drugs and proteins. This method enables researchers to simulate how a small molecule binds to a biological target, allowing for the examination and analysis of the stability and strength of these interactions. Molecular docking typically involves two main stages: spatial searching to identify potential binding positions and energy evaluation to determine the stability of these positions. This technique is widely utilized in drug discovery and the design of new molecules, facilitating the identification of promising compounds for disease treatment. By employing molecular docking, researchers can gain a deeper understanding of the mechanisms of action of drugs and proteins, enabling necessary optimizations to enhance efficacy and reduce side effects. In the present study, we focused on MTH1, for which we obtained the crystallographic structure from the Protein Data Bank (PDB) under ID 4N1U [26]. The amino acid residues critical for the catalytic site were delineated, and a docking protocol was established to predict bioactive conformations. As demonstrated in Fig 6, the root mean square deviation (RMSD) between the native ligand (yellow) and the re-docked ligand (red) was quantified at 0.75 Å, thereby affirming the validity of the docking protocol [27].

**Fig 7:**
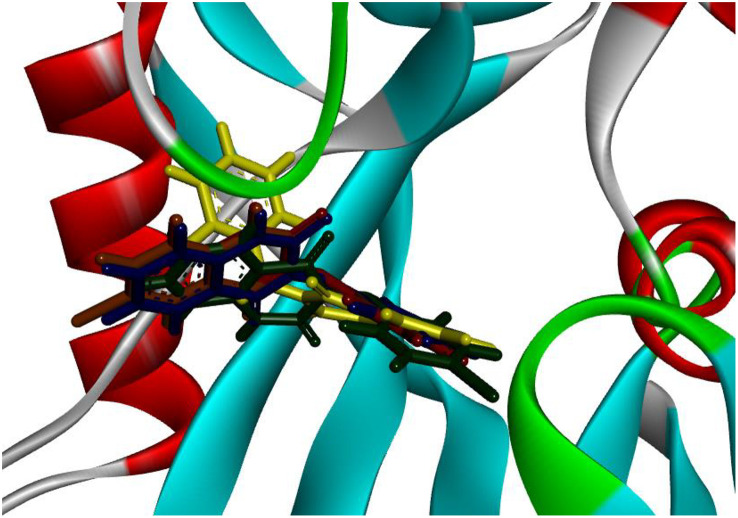
Superimposed structures of synthesized derivatives (3a − e) in active site of 4N1U.

Fig 7 demonstrates that all derivatives of 3-(arylamino) quinazoline-2,4(1*H*,3*H*)-diones **(3a–e)** studied engaged with the identical binding site as the native compound. To evaluate the binding affinities and scores of these derivatives, the interactions between the synthesized compounds and the amino acid residues located in the active site were analyzed.

The results indicate that all derivatives displayed superior binding scores and notable binding affinities. The amino acid residues involved in both covalent and non-covalent interactions with the 3-(arylamino) quinazoline-2,4(1*H*,3*H*)-dione derivatives **(3a–e)** include Phe139, Phe74, Phe35, Gly34, Gly36, Glu56, Glu100, Met101, Asn33, Trp117, Phe27, Val83, Phe72, Met81, Trp123, Leu9, Lys23, Lys38, Ile70, Tyr7, Gly37, Asp119, Asp120, Thr8, and Gly28. An analysis of Table 4 offers insights into the docking scores and types of interactions for the 3-(arylamino) quinazoline-2,4(1*H*,3*H*)-dione derivatives **(3a–e)** at the active site of MTH1, highlighting their binding affinities and interaction profiles.

**Table 4:** Docking scores and interaction for each 3-(arylamino) quinazoline-2,4(1*H*,3*H*)-dione derivatives (3a − e).

Types of interactions												
Compound	Docking Score (kcal/mol)	MM-GBSA dG bind	Conventional Hydrogen Bond	Pi-donor hydrogen Bond	Carbon Hydrogen Bond	Pi-Alkyl	Alkyl	Pi-Pi Stacked	Pi-Pi T-shaped	Pi-Sulfur	Pi-Lone pair	Van der Waals
**3a**	-6.77	-46.96	Phe27, Trp117, Asn33	Asn33		Val83		Trp117	Phe27			Lys38, Tyr7, Gly37, Met81, Asp119, Phe72, Ile70, Trp123, Leu9, Gly28, Lys23
**3b**	-6.91	-44.97	Asn33, Trp117			Phe72, Phe139, Phe74, Met81, Trp117		Trp117				Lys23, Gly28, Phe27, Asp119, Trp123, Asp120, Leu9, Val83, Thr8, Tyr7, Gly37, Lys38
**3c**	-6.67	-49.83	Phe27, Trp117, Asn33	Asn33		Val83	Lys38	Trp117	Phe27			Gly37, Tyr7, Met81, Asp119, Phe72, Trp123, Ile70, Leu9, Gly28, Lys23
**3d**	-7.24	-53.40	Phe27, Asn33, Trp117			Trp123, Val83, Phe72	Val83,Ile70,Leu9	Trp117				Lys38, Tyr7, Gly37, Met81, Asp119, Asp120, Gly28, Lys23
**3e**	-5.77	-39.75				Trp123, Phe72, Leu9, Val83, Lys23	Ile70,Leu9,Val83		Phe72	Met101	Gly36	Asp120, Asp119, Trp117, Asn33, Met81, Phe27, Phe35, Gly34, Glu56, Glu100, Gly37, Tyr7, Thr8
**BAY 707**	-6.32	-35.55	Asp119	Asn33	Thr8, Phe27, Asp119	Leu9, Val83, Phe27,Trp117, Phe72, Phe74, Phe139	Lys23		Phe72	Met81		Gly36, Gly28, Gly37, Tyr7, Gln142, Pro118

**Fig 8:**
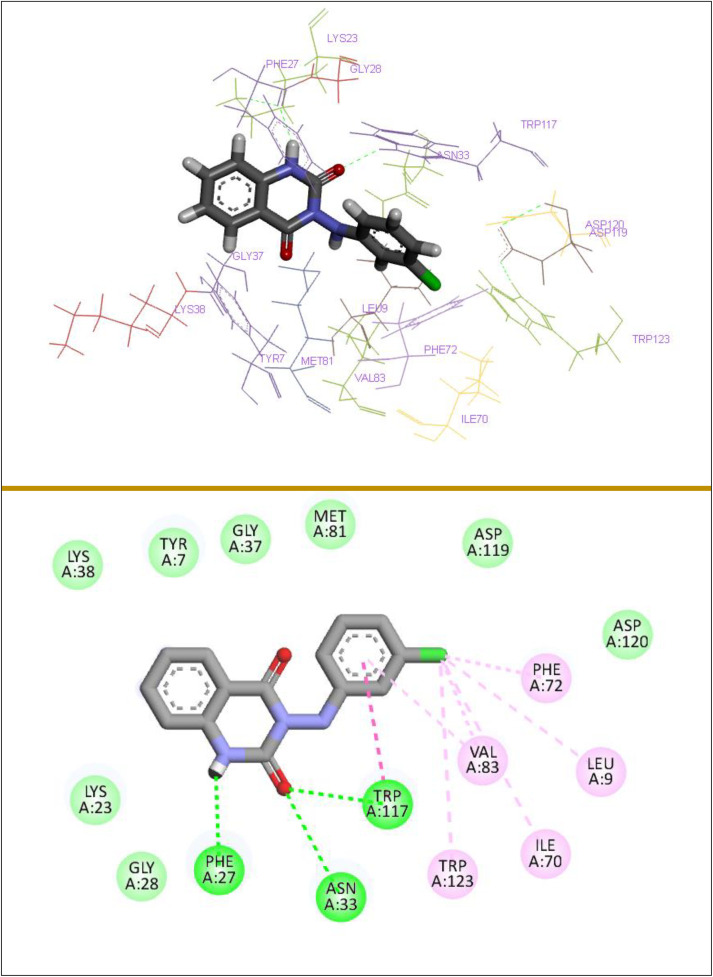
3D and 2D binding interactions of (3d) with targeted MTH1 (PDB: 4N1U).

Compound **(3d)** stands out with the highest docking score of −7.24, indicating a strong binding affinity, primarily due to its extensive hydrogen bonding interactions with residues such as Phe27, Trp117, and Asn33. Additionally, it forms significant pi-alkyl interactions with Trp123, Val83, Phe72, and alkyl and pi-pi stacked interactions with Val83, Ile70, Leu9, and Trp117, which collectively enhance its stability in the binding site. In comparison, compound **(3b)**, with a docking score of −6.91, also demonstrates a robust interaction profile, featuring multiple hydrogen bonds and pi-alkyl interactions with residues like Phe72, Phe139, Phe74, and Met81, although it lacks the overall binding stability observed in **(3d)**. Compound **(3a)**, scoring −6.77, shares similar hydrogen bonding interactions but exhibits fewer pi-pi interactions, which may contribute to its slightly lower binding affinity. Compound **(3c)**, with a score of −6.67, shows a comparable interaction pattern but is less effective due to a reduced number of hydrophobic interactions. Conversely, compound **(3e)**, with the lowest docking score of −5.77, reveals limited interactions and a weaker binding profile, primarily due to fewer hydrogen bonds and hydrophobic contacts, indicating that its structural characteristics may not favor effective binding to the MTH1 active site. All synthesized compounds exhibit binding affinities attributable to the presence of crucial hydrogen bonds, alongside extensive hydrophobic and van der Waals interactions. These factors contribute to their stabilization within the binding pocket, underscoring the importance of the diversity and strength of these interactions. Fig 8 illustrates the detailed 3D and 2D binding interactions of compound **(3d)** within the active site of MTH1. A comprehensive understanding of these interactions could yield critical insights for the development of new inhibitors that utilize comparable binding mechanisms, suggesting that compounds with a more advantageous interaction profile are likely to serve as better candidates for therapeutic targeting of MTH1.

**Fig 9:**
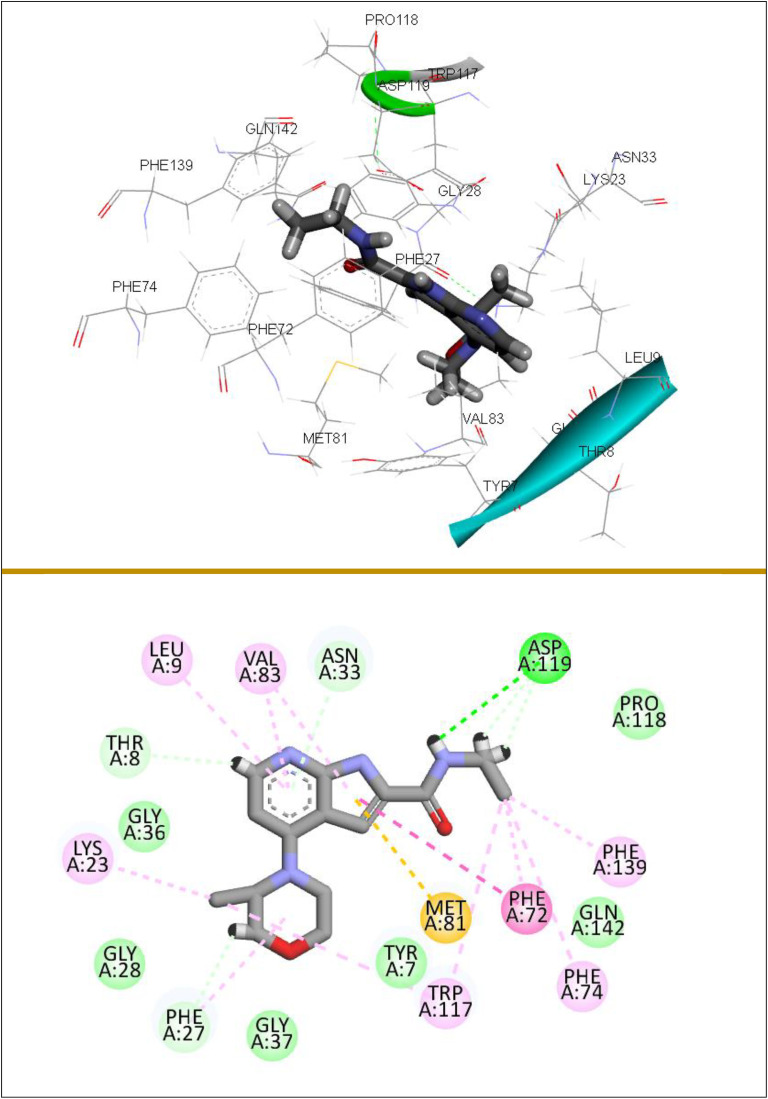
3D and 2D binding interactions of BAY-707 with targeted MTH1.

BAY-707 is a potent and selective inhibitor of MTH1, designed to target the enzyme’s active site and impede its ability to hydrolyze oxidized nucleotides, specifically 8-oxodGTP. By inhibiting MTH1, BAY-707 leads to an accumulation of these harmful oxidized nucleotides within the cell, resulting in increased oxidative stress and DNA damage. This mechanism is particularly effective in cancer cells, which often rely on MTH1 to maintain genomic stability and replicate efficiently despite elevated oxidative conditions. Consequently, the use of BAY-707 not only disrupts the normal nucleotide pool dynamics but also enhances the vulnerability of tumor cells to DNA-damaging agents, making it a promising candidate for combination therapies aimed at improving cancer treatment outcomes. We utilized BAY-707 as a reference compound to compare the inhibitory effects of 3-(arylamino) quinazoline-2,4(1*H*,3*H*)-dione derivatives **(3a − e)** on MTH1. The 3D and 2D interactions of BAY-707 are shown in Fig 9. BAY-707, with a docking score of −6.32 kcal/mol, exhibits multiple interactions, including hydrogen and C–hydrogen bonding with the amino acid residues Asp119, Thr8, Phe27, and Asn33. Additionally, residues such as Lys23, Leu9, Val83, Trp117, Phe72, Phe74, and Phe139 are also involved in the interaction network, indicating a well-coordinated binding environment that enhances the efficacy of BAY-707 as an MTH1 inhibitor. However, all 3-(arylamino) quinazoline-2,4(1*H*,3*H*)-dione derivatives **(3a − e)**, except for compound **(3e)**, demonstrated superior docking scores compared to BAY-707. This finding indicates the remarkable effectiveness of these 3-(arylamino) quinazoline-2,4(1*H*,3*H*)-dione derivatives **(3a − e)** as potential inhibitors of MTH1, suggesting their promise for further development in therapeutic applications targeting this enzyme.

### Binding free energy calculation

The MM-GBSA model is utilized to evaluate the absolute binding affinities of ligand-protein complexes by estimating their binding free energy. The calculations were conducted using the MM-GBSA panel of the Prime module, with all parameters maintained at their default configurations [28]. The formula employed for the computation of MM-GBSA is as follows:


$${\Delta G}_{\left(Binding\right)}={\Delta E}_{\left(MM\right)}\;+{\Delta G}_{\left(SA\right)}\;+{\Delta G}_{\left(Solvation\right)}$$

Where:

signifies the disparity in nonpolar solvation energies between the MTH-inhibitor complex and the cumulative surface area energies of the unbound MTH1 enzyme and the inhibitor. indicates the variation in molecular mechanics energies of the MTH1-inhibitor complex in comparison to the aggregated minimized energies of the unbound components, specifically the MTH1 enzyme and the inhibitor. denotes the difference in polar solvation energies between the MTH1-inhibitor complex and the individual unbound entities. The binding affinity of ligands to the receptor was evaluated using MM-GBSA-based rescoring, which provides a more accurate estimation of their binding free energies. In this study, a series of 3-(arylamino)quinazoline-2,4(1*H*,3*H*)-dione derivatives **(3a − e)** were screened and compared to BAY 707, which served as a reference compound. The calculated binding free energies for these derivatives and BAY 707 ranged from −36.75 to −53.66 kcal/mol. The MM-GBSA results showed a strong correlation with the initial docking scores. Notably, derivative **(3d)** exhibited the most favorable binding free energy, indicating the strongest predicted binding affinity among all the compounds. Additionally, apart from derivative **(3e)**, the other compounds demonstrated improved binding affinities compared to BAY 707, highlighting their potential as more effective receptor binders.

### MD Simulation

**Fig 10:**
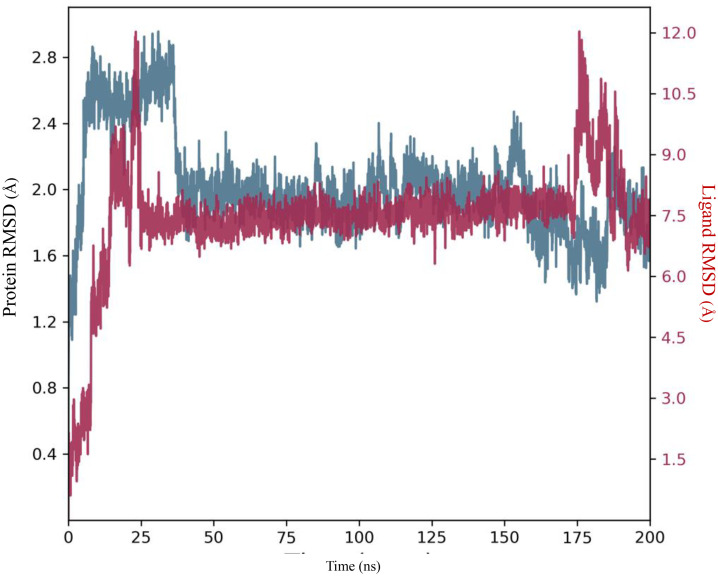
RMSD of the 3RKU–(3d) complex during a 200 ns MD simulation.

The plot showing the RMSD for a protein-ligand complex over a 200-ns MD simulation provides important insights into the structural stability of both the protein and the ligand, as shown in Fig 10. Initially, the plot shows fluctuations in both protein and ligand RMSD during the first 25 ns, suggesting some instability as the system adjusts to the simulated environment. After approximately 25 ns, both RMSD values stabilize, indicating that the system has reached an equilibrium state where structural changes are minimal. The protein RMSD remains relatively stable, hovering around 1.5 to 2.5 Å after the initial fluctuations, demonstrating that the protein structure maintains its integrity throughout the simulation. In contrast, the ligand RMSD exhibits more significant fluctuations, especially towards the end of the simulation around 175 ns, indicating that the ligand experiences more movement or interactions with the protein, which may suggest binding or unbinding events. Overall, the oscillations in the ligand RMSD, particularly at the end, could reflect the ligand adjusting its position due to interactions with the protein or conformational changes in the binding site.

**Fig 11:**
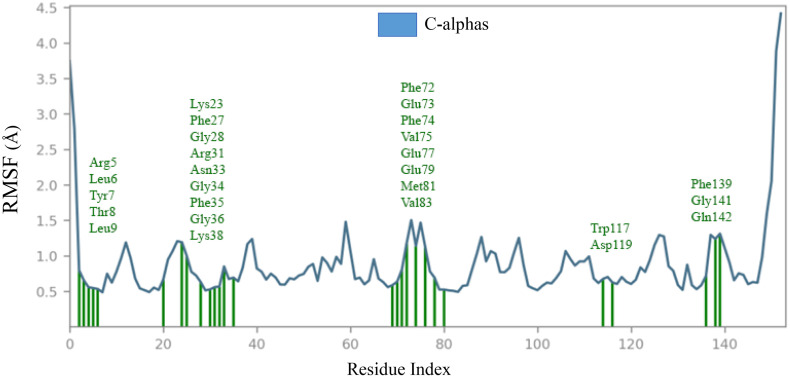
RMSF of the 4N1U–(3d) complex during a 200 ns MD simulation.

The plot of root mean square fluctuation (RMSF) for a protein during a MD simulation offers valuable insights into the flexibility and stability of various residues. The RMSF values range from approximately 0.5 Å to 4.5 Å, indicating significant variations in the mobility of different regions within the protein. As shown in Fig 11, some residues exhibit elevated RMSF values, suggesting that these regions are highly dynamic and may play a critical role in interactions or conformational changes. Conversely, residues with lower RMSF values display enhanced stability and rigidity within the protein structure. The green lines in the RMSF plot denote specific residues that exhibit significant fluctuations during the MD simulation. The residues associated with these green lines include Arg5, Leu6, Tyr7, Thr8, Leu9, Lys23, Phe27, Gly28, Arg31, Asn33, Gly34, Phe35, Gly36, Lys38, Phe72, Glu73, Phe74, Val75, Glu77, Glu79, Met81, Val83, Trp117, Asp119, Phe139, Gly141, and Gln142. These residues are implicated in various types of interactions, underscoring their significance in the binding affinity of the analyzed compounds.

**Fig 12:**
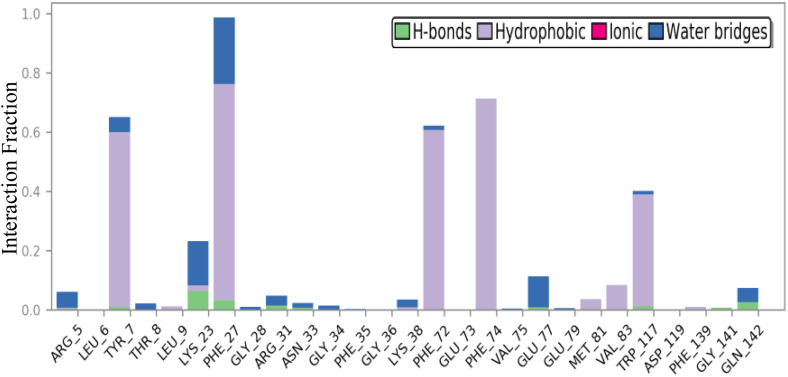
Interaction fraction of the 4N1U–(3d) complex during a 200 ns MD simulation.

The bar chart in Fig 12 provides a detailed representation of the interaction fractions between specific amino acid residues and the ligand throughout the MD simulation. The interactions are categorized into four distinct types: Hydrogen Bonds (green), Hydrophobic Interactions (purple), Ionic Interactions (light blue), and Water Bridges (dark blue). Notably, residues such as Lys23, Phe27, Arg131, and Gln142 exhibit hydrogen bond interactions, with Lys23 showing the highest fraction, indicating its critical role in stabilizing the protein-ligand complex. Conversely, residues such as Tyr7, Phe27, Phe72, Phe74, and Trp117 demonstrate significant hydrophobic interactions, suggesting that these regions contribute to the overall binding affinity through non-polar interactions. The presence of ionic interactions is minimal, with no notable contributions displayed. Additionally, the chart emphasizes the importance of water bridges, particularly involving residues such as Lys23 and Phe27, which may facilitate the stabilization of ligand binding through solvent-mediated interactions. Overall, this analysis underscores the complexity of protein-ligand interactions, elucidating the diverse mechanisms by which binding affinity is attained and maintained.

**Fig 13:**
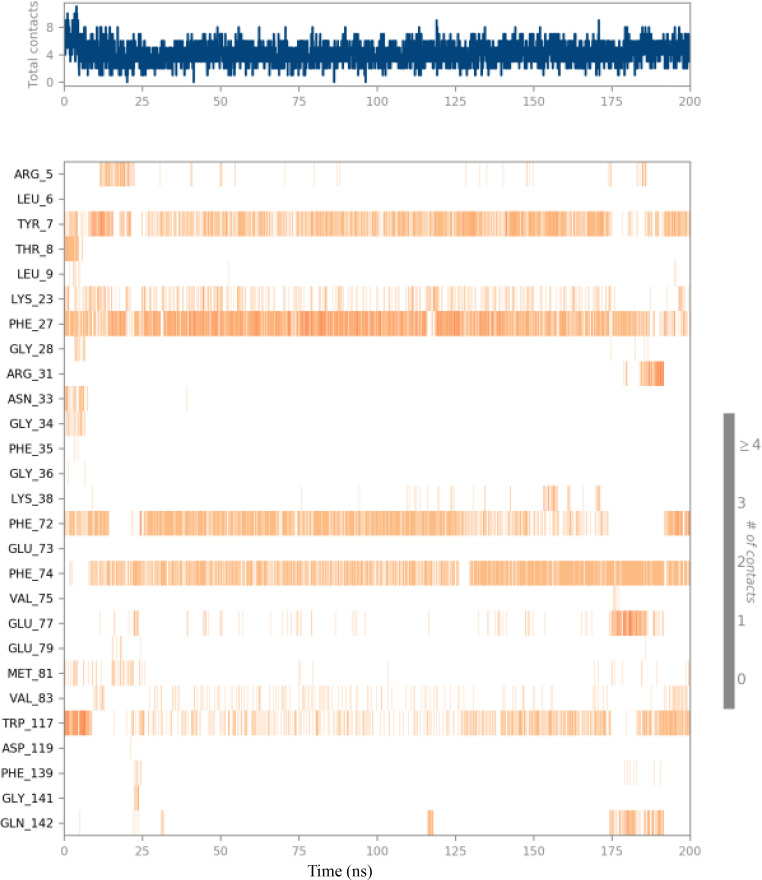
Timeline representation showing the different contacts formed by the 4N1U–(3d) complex during a 200 ns MD simulation.

The investigation into the entrapment of compounds within specific amino acid residues provides valuable insights into the binding mechanism. As depicted in Fig 13, residues Tyr7, Lys23, Phe27, Phe72, Phe74, and Trp117 remained consistently encapsulated by compound **(3d)** throughout the majority of the simulation duration. In contrast, other amino acid residues displayed a lower degree of entrapment, indicating that their interactions with the ligand occurred with reduced frequency. These findings underscore the significance of particular residues in the binding process, suggesting that certain amino acids play a more pivotal role in stabilizing the ligand within the binding pocket. A comprehensive understanding of the dynamics of these interactions contributes to elucidating the binding mechanism and can guide the design of more effective compounds targeting specific proteins.

**Fig 14:**
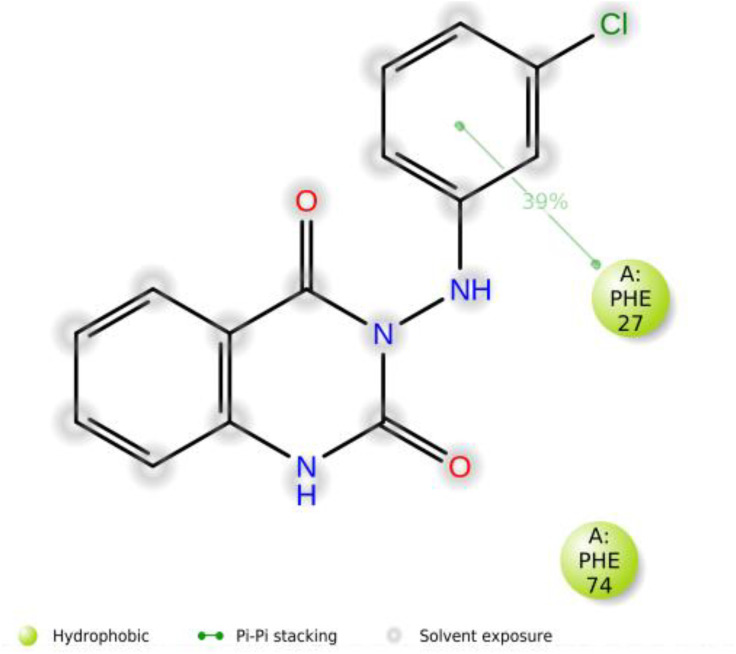
2D interaction diagram of compound (3d) in complex with 4N1U during a 200 ns MD simulation.

The 2D trajectory interaction diagram presented in Fig 14 highlights significant residues, including Phe27 and Phe74. Residue Phe27 exhibits a 39% interaction frequency, indicating its important hydrophobic role through a Pi-Pi stacked interaction that stabilizes the ligand. This interaction is preserved throughout the MD trajectory, further emphasizing its critical importance in the binding process.

**Fig 15:**
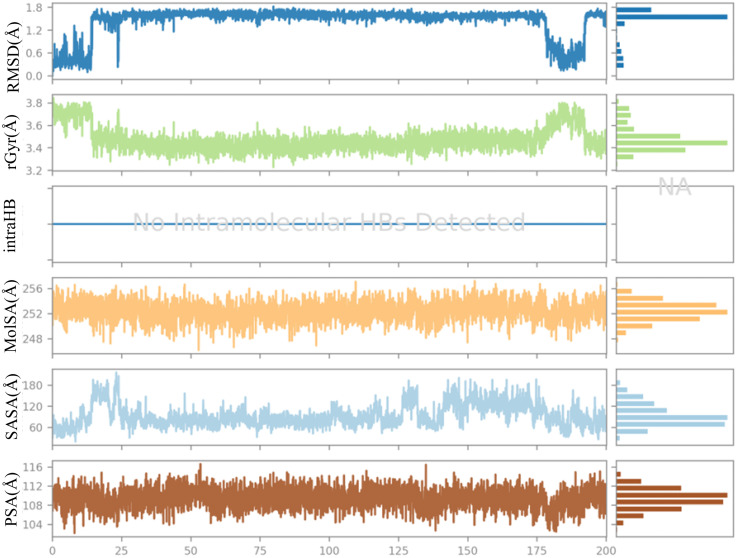
Ligand properties of compound (3d) in complex with 4N1U during 200 ns MD simulation.

The examination of the structural and dynamic characteristics of molecules is crucial for a thorough understanding of their behavior across different conditions. Among the primary analytical tools utilized in this context is the RMSD, which assists in evaluating the stability and structural alignment of molecules. The radius of gyration (rGyr) provides insights into the mass distribution within the molecule, shedding light on its shape and degree of compactness. The solvent-accessible surface area (SASA) measures the portion of the molecule’s surface that is accessible to solvent, thereby enhancing the understanding of intermolecular interactions. Intramolecular hydrogen bonds (intraHB) are vital for maintaining the stability of the 3D configurations of molecules. Moreover, the molecular surface area (MolSA) and polar surface area (PSA) define the surface characteristics of the molecule, which significantly affect its biological and chemical interactions. Together, these parameters are essential for analyzing and predicting molecular behavior in MD simulations. Fig 15 provides a detailed overview of these properties for the **(3d)**-4N1U complex. The RMSD values stabilize after an initial fluctuation, indicating that the ligand reaches a stable conformation around 1.0 Å, suggesting that the ligand maintains its structural integrity throughout the simulation. The rGyr values show minor fluctuations, indicating that the ligand remains relatively stable in terms of its spatial distribution, reflecting a consistent conformation during the simulation. Notably, no intraHB were identified during the simulation, indicating that the ligand does not establish significant internal interactions that could further stabilize its structure. The MolSA displayed variability between 241 and 259 Å², while the SASA fluctuated from 10 to 210 Å², and the PSA ranged between 102 and 117 Å².

### Drug-likeness prediction

Drug-likeness is a fundamental principle in drug discovery that evaluates the potential of compounds to become effective pharmaceutical agents. Several criteria and guidelines have been established to assess drug-likeness, including Lipinski’s RO5, which posits that compounds should ideally possess a molecular weight below 500 daltons, a logP value not exceeding 5, no more than 5 hydrogen bond donors, and a maximum of 10 hydrogen bond acceptors. Ghose’s rule further refines these criteria by incorporating additional parameters such as molar refractivity and the number of rotatable bonds, while Veber’s rule emphasizes the significance of PSA and rotatable bonds in predicting bioavailability. Egan’s rule also contributes to this framework by focusing on the balance between hydrophilicity and lipophilicity. Furthermore, pan assay interference compounds (PAINS) represent a critical concept in medicinal chemistry, aimed at identifying compounds that may exhibit non-specific activity or interfere with biological assays. These compounds frequently possess structural characteristics that lead to erroneous results in high-throughput screening, such as reactive functional groups or specific molecular scaffolds that can induce false positives. The importance of PAINS lies in their capacity to assist researchers in filtering out potentially problematic candidates early in the drug discovery process. By recognizing and circumventing these compounds, scientists can enhance the reliability of their screening outcomes and concentrate on more promising drug candidates with a higher probability of success in subsequent development phases. Consequently, the integration of PAINS alerts into the drug discovery workflow is essential for improving the quality and efficacy of therapeutic agents. The analysis of the predicted physicochemical and drug-likeness properties of 3-(arylamino) quinazoline-2,4(1*H*,3*H*)-dione derivatives **(3a–e)** reveals several noteworthy characteristics that support their potential as viable pharmaceutical agents. As shown in Table 5, all 3-(arylamino) quinazoline-2,4(1*H*,3*H*)-dione derivatives **(3a–e)** meet the drug-likeness criteria established by Lipinski, Ghose, and Veber, indicating their potential as viable pharmaceutical agents. This adherence is a significant strength, as it highlights that these synthesized ligands exhibit properties similar to those of BAY 707. Importantly, the absence of PAINS or Brenk alerts across all 3-(arylamino) quinazoline-2,4(1*H*,3*H*)-dione derivatives **(3a–e)** underscores their safety in drug design, which is crucial for any therapeutic candidate.

**Table 5:** Predicted Physicochemical and Drug-Likeness Properties of the 3-(arylamino) quinazoline-2,4(1*H*,3*H*)-dione derivatives (3a − e).

Compound	MW (g/mol)	HBA	HBD	TPSA	MLog P	MR	nRot	Lipinski	Ghose	Veber’s rule	Egan	PAINS (alert)	Brenk
**3a**	253.26	2	2	66.89	2.63	74.11	2	Yes	Yes	Yes	Yes	0	0
**3b**	332.15	2	2	66.89	3.01	81.81	2	Yes	Yes	Yes	Yes	0	0
**3c**	332.15	2	2	66.89	3.01	81.81	2	Yes	Yes	Yes	Yes	0	0
**3d**	287.70	2	2	66.89	2.88	79.12	2	Yes	Yes	Yes	Yes	0	0
**3e**	287.70	2	2	66.89	2.88	79.12	2	Yes	Yes	Yes	Yes	0	0
**BAY 707**	288.34	3	2	70.25	0.79	84.43	4	Yes	Yes	Yes	Yes	0	0

### In *Silico* ADMET prediction

ADMET constitutes a fundamental framework in pharmaceutical development that evaluates the pharmacokinetic and pharmacodynamic properties of prospective therapeutic agents. Comprehending ADMET characteristics is imperative for forecasting a compound’s behavior within biological systems, thereby influencing its efficacy and safety profile. Absorption denotes the extent and rate at which a drug permeates systemic circulation, while distribution investigates the manner in which the drug disseminates throughout bodily tissues. Metabolism encompasses the biochemical conversion of the drug, frequently impacting its bioactivity and elimination. Excretion pertains to the clearance of the drug and its metabolites from the organism, primarily via renal or hepatic pathways. Toxicity assessment is vital for identifying deleterious effects that may emerge from drug exposure. A thorough ADMET evaluation not only facilitates the optimization of lead compounds during the drug discovery continuum but also augments the probability of favorable clinical outcomes by mitigating the risk of failure due to adverse pharmacokinetic or toxicological profiles. The assessment of the pharmacokinetic properties of 3-(arylamino) quinazoline-2,4(1*H*,3*H*)-dione derivatives **(3a–e)** was conducted utilizing the pkCSM database [29]. As illustrated in Table 6, all 3-(arylamino) quinazoline-2,4(1*H*,3*H*)-dione derivatives **(3a-e)** and BAY 707 demonstrate high percentages of HIA. BAY 707 exhibits a slightly higher absorption percentage of 96.307%, whereas the values for the compounds 3a-e range from 93.333% to 94.868%. This suggests that these compounds possess favorable intestinal absorption characteristics. Regarding distribution, the volume of distribution (VDss) values are negative for both groups, indicating limited distribution into tissues. BAY 707 has a VDss of −0.419 L/kg, which falls within the range of the 3-(arylamino) quinazoline-2,4(1*H*,3*H*)-dione derivatives **(3a-e)** (−0.583 to −0.516 L/kg). However, a notable distinction arises in permeability across the blood-brain barrier (BBB). BAY 707 has a negative logBB value of −1.260, indicating poor permeability through the BBB. In contrast, the 3-(arylamino) quinazoline-2,4(1*H*,3*H*)-dione derivatives **(3a-e)** possess positive logBB values (ranging from 0.090 to 0.172), suggesting that these compounds have better BBB permeability than BAY 707. Additionally, BAY 707 shows significantly lower logPS values (−3.753), indicating inadequate permeability to the central nervous system (CNS), while the 3-(arylamino) quinazoline-2,4(1*H*,3*H*)-dione derivatives **(3a-e)** demonstrate higher logPS values (ranging from −2.084 to −2.224), suggesting improved CNS permeability. In terms of metabolism, all 3-(arylamino) quinazoline-2,4(1*H*,3*H*)-dione derivatives **(3a-e)** are predicted to be substrates for major cytochrome P450 enzymes, suggesting substantial metabolic processing, while BAY 707 is not predicted to be a substrate for these enzymes. Regarding excretion, the clearance values for the compounds vary. BAY 707 has a predicted clearance of 0.576 mL/min/kg. In the context of toxicity, none of the 3-(arylamino) quinazoline-2,4(1*H*,3*H*)-dione derivatives **(3a-e)** or BAY 707 are predicted to be AMES toxic. However, BAY 707 is anticipated to exhibit hepatotoxicity, whereas the synthesized compounds **(3a-e)** are not predicted to have such effects.

**Table 6:** Predicted Pharmacokinetic (ADMET) Properties of the 3-(arylamino) quinazoline-2,4(1*H*,3*H*)-dione derivatives (3a − e).

Compound	Absorption		Distribution					Metabolism				Excretion	Toxicity	
	HIA	VDss	BBB permeability	CNS permeability	substrate		inhibitors					total clearance	AMES toxicity	Hepatotoxicity
					2D6	3A4	1A2	2C19	2C9	2D6	3A4			
	Numeric (% Absorbed)	Numeric (L kg^-1^)	Numeric (log BB)	Numeric (log PS)				Categorical (Yes/No)				Numeric (mL min^ − 1^ kg^ − 1^)	Categorical (Yes/No)	
**3a**	94.868	−0.583	0.172	−2.224	Yes	Yes	Yes	Yes	No	No	Yes	0.520	No	No
**3b**	93.416	−0.549	0.107	−2.090	Yes	Yes	Yes	Yes	No	No	Yes	−0.166	No	No
**3c**	93.333	−0.546	0.127	−2.084	Yes	Yes	Yes	Yes	No	No	Yes	−0.105	No	No
**3d**	93.993	−0.516	0.090	−2.109	Yes	Yes	Yes	Yes	No	No	No	−0.081	No	No
**3e**	94.137	−0.525	0.169	−2.111	Yes	Yes	Yes	Yes	No	No	No	0.046	No	No
**BAY 707**	96.307	−0.419	−1.260	−3.753	No	No	Yes	No	No	No	No	0.576	No	Yes

## Conclusion

We have successfully designed and synthesized a novel class of 3-(arylamino) quinazoline-2,4(1*H*,3*H*)-dione derivatives utilizing TBHP and iodine as reagents. To investigate the biological potential of these compounds, we conducted a computational study. The docking results indicated that all synthesized compounds exhibit high binding affinities for the MTH1 enzyme. In comparison to the reference compound BAY-707, all compounds except compound **(3e)** showed better docking scores, among which compound **(3d)** exhibited the highest binding affinity. Notably, the results from MM-GBSA rescoring were consistent with the docking outcomes, confirming that the key interactions predicted in both approaches were in agreement. This concordance further supports the potential of these compounds as effective binders for the MTH1 enzyme. The RMSD analysis demonstrated that compound **(3d)** consistently maintained stable interactions with key residues of MTH1, indicating that the **(3d)**-MTH1 complex remained thermodynamically stable throughout the 200 ns trajectory. DFT analysis confirmed that all of these molecules possess high reactivity. In particular, compound **(3c)** exhibited the lowest energy gap between the HOMO and LUMO, which correlates with the compound’s stability and biological activity. Furthermore, the analysis of ADMET properties revealed that none of the compounds violate Lipinski’s RO5 or the Ghose, Veber, and Egan rules. The physiological profiling indicated that all compounds have potential for HIA and demonstrate no liver toxicity, whereas BAY-707 is expected to exhibit hepatotoxicity. The results of the chemo-informatics study would benefit from further validation through additional in *vivo* and in *vitro* research.

## Experimental

### General information

All solvents and reagents were purchased from Aldrich and Merck Chemical Co. DMSO- *d*_*6*_ was used as the solvent to obtain NMR spectra on a Bruker (300 MHz for ^1^H and 75 MHz for ^13^C). Melting points were measured using an Electrothermal 9100. An Agilent 5975C VL MSD with a triple-axis detector recorded mass spectra at 70 eV. IR spectra were measured using the Bruker Tensor 27.

### General method for synthesizing 3-(arylamino) quinazoline-2,4(1*H*,3*H*)-dione derivatives (3a − e)

Derivatives of isatin **(1)**, (1 mmol), phenyl hydrazine **(2)**, 1 mmol), iodine (0.3 mmol), and TBHP, 70% aqueous solution, (1 mmol) were combined with MeOH (10 mL) in a round-bottom flask fitted with a condenser. The reaction mixture was stirred and heated under reflux for 5 hours, with the reaction progress monitored by thin-layer chromatography (TLC) using a 1:1 solvent system of ethyl acetate and n-hexane. Upon completion of the reaction, the mixture underwent vacuum evaporation, followed by dilution with ethyl acetate and washing with a sodium thiosulfate (Na₂S₂O₃) solution. The organic layer was then washed with brine, dried over anhydrous Na₂SO₄, and concentrated under reduced pressure. The resulting compounds **(3a–e)** were purified through standard column chromatography on silica gel (60–120 mesh) utilizing a gradient solvent system of *n*-hexane and ethyl acetate.

### 3-(phenylamino)quinazoline-2,4(1*H*,3*H*)-dione (3a)

White solid; yield: 70%; mp: 224−226°C; IR (KBr): 3442, 3277 (N-H), 1739, 1668 (C = O), 1276, 1239 and 1163 cm^-1^ (C-N); ^1^H NMR (300 MHz, DMSO-*d*_*6*_): δ ppm, 11.65 (*s*, 1H, H-1), 8.49 (*s*, 1H, NHPh), 7.96 (*d*, 1H, *J* = 6.8 Hz, H-5), 7.71 (*t*, 1H, *J* = 7.2 Hz, H-7), 7.25 (*t*, 1H, *J* = 7.2 Hz, H-6), 7.24 (*d*, 1H, *J* = 8.0 Hz, H-8), 7.15 (*t*, 2H, *J* = 7. 8 Hz, H-3’, 5’), 6.77 (*t*, 1H, *J* = 7.4 Hz, H-4’), 6.66 (*d*, 2H, *J* = 8.0 Hz, H-2’, 6’); ^13^C NMR (75 MHz, DMSO-*d*_*6*_): δ ppm, 162 (C-4), 150.4 (C-2), 147.8 (C-1’), 139.8 (C-8a), 135.8 (C-7), 129.3 (C-2’, 6’), 128.1 (C-4’), 123.2 (C-5), 119.8 (C-6), 115.9 (C-8), 114.5 (C-4a), 112.8 (C-3’, 5’); MS (EI, 70 eV): *m/z* (%) = 253 (100) [M] ^+^, 209 (1), 162 (5), 146 (72), 119 (16), 92 (26), 91 (25), 90 (14), 64 (13), 63 (9).

### 3-(4-bromophenylamino)quinazoline-2,4(1*H*,3*H*)-dione (3b)

White solid; yield: 40%; mp: 249−251°C; IR (KBr): 3299, 3204, 3145 (N-H), 1730, 1671 (C = O), 1280, 1242 and 1157 cm^-1^ (C-N); ^1^H NMR (300 MHz, DMSO-*d*_*6*_): δ ppm, 11.52 (*s*, 1H, H-1), 8.65 (*s*, 1H, NHPh), 7.96 (*dd*, 1H, *J* = 8.2, 1.4 Hz, H-5), 7.71 (*dt*, 1H, *J* = 7.7, 1.4 Hz, H-7), 7.25 (*t*, 1H, *J* = 7 Hz, H-6), 7.24 (*d*, 1H, *J* = 6.8 Hz, H-8), 7.18 (*d*, 2H, *J* = 9.4, 2.2 Hz, H-3’, 5’), 6.7 (*d*, 2H, *J* = 9.4, 2.6 Hz, H-2’, 6’); ^13^C NMR (75 MHz, DMSO-*d*_*6*_): δ ppm, 161.9 (C-4), 150.3 (C-2), 146.8 (C-1’), 139.8 (C-8a), 135.8 (C-7), 129.0 (C-2’, C6’), 128.1 (C-4’), 123.2 (C-5), 123.2 (C-6), 115.9 (C-8), 114.5 (C-4a), 114.4 (C-3’, C5’).

### 6-bromo-3-(phenylamino)quinazoline-2,4(1*H*,3*H*)-dione (3c)

White solid; yield: 30%; mp: 257−259°C; IR (KBr): 3425, 3270 (N-H), 1740, 1666 (C = O), 1271 and 1167 cm^-1^ (C-N); ^1^H NMR (300 MHz, DMSO-*d*_*6*_): δ ppm, 11.8 (*s*, 1H, H-1), 8.50 (*s*, 1H, NHPh), 7.90 (*d*, 1H, *J* = 2.4 Hz, H-5), 7.77 (*dd*, 1H, *J* = 8.6, 2.6 Hz, H-7), 7.26 (*d*, 1H, *J* = 8.4 Hz, H-8), 7.15 (*t*, 2H, *J* = 7.8, 1.2 Hz, H-3’, 5’), 6.78 (*t*, 1H, *J* = 7.4 Hz, H-4’), 6.68 (*d*, 2H, *J* = 7.6 Hz, H-2’, 6’); ^13^C NMR (75 MHz, DMSO-*d*_*6*_): δ ppm, 161.1 (C-4), 150.2 (C-2), 147.6 (C-1’), 138.7 (C-8a), 135.6 (C-7), 129.3 (C-2’, 6’), 127.1 (C-4’), 127.0 (C-5), 119.9 (C-6), 118.2 (C-8), 115.0 (C-4a), 112.8 (C-3’, 5’).

### 3-(3-chlorophenylamino)quinazoline-2,4(1*H*,3*H*)-dione (3d)

White solid; yield: 60%; mp: 290−292°C; IR (KBr): 3428, 3321, 3147 (N-H), 1728, 1671 (C = O), 1277, 1238 and 1156 cm^-1^ (C-N); ^1^H NMR (300 MHz, DMSO-*d*_*6*_): δ ppm, 11.63 (*s*, 1H, H-1), 8.74 (*s*, 1H, NHPh), 7.93 (*dd*, 1H, *J* = 8.1, 1.2 Hz, H-5), 7.69 (*dt*, 1H, *J* = 7.2, 1.4 Hz, H-7), 7.23 (*t*, 1H, *J* = 7.6 Hz, H-6), 7.22 (*d*, 1H, *J* = 7.8 Hz, H-8), 7.14 (*t*, 1H, *J* = 6.8 Hz, H-5’), 6.77 (*d*, 1H, *J* = 8.3 Hz, H-4’), 6.70 (*s*, 1H, H-2’), 6.63 (*d*, 1H, *J* = 8.1 Hz, H-6’); ^13^C NMR (75 MHz, DMSO-*d*_*6*_): δ ppm, 161.9 (C-4), 150.3 (C-2), 149.4 (C-1’), 139.8 (C-8a), 135.8 (C-7), 134.0 (C-2’), 130.9 (C-6’), 128.1 (C-4’), 123.1 (C-5), 119.3 (C-6), 115.9 (C-8), 114.5 (C-4a), 112.2 (C-3’), 111.4 (C-5’).

### 8-chloro-3-(phenylamino)quinazoline-2,4(1*H*,3*H*)-dione (3e)

White solid; yield: 20%; mp: 242−244°C; IR (KBr): 3427, 3305, 3267 (N-H), 1724, 1672 (C = O), 1261 and 1161 cm^-1^ (C-N); ^1^H NMR (300 MHz, DMSO-*d*_*6*_): δ ppm, 11.19 (*s*, 1H, H-1), 8.52 (*s*, 1H, NHPh), 7.94 (*dd*, 1H, *J* = 7.9, 1.4 Hz, H-5), 7.84 (*dd*, 1H, *J* = 7.9, 2.4 Hz, H-7), 7.24 (*t*, 1H, *J* = 7.8 Hz, H-6), 7.13 (*t*, 2H, *J* = 7.6, 1.2 Hz, H-3’, 5’), 6.75 (*t*, 1H, *J* = 7.2 Hz, H-4’), 6.68 (*d*, 2H, *J* = 8.0 Hz, H-2’, 6’); ^13^C NMR (75 MHz, DMSO-*d*_*6*_): δ ppm, 161.9 (C-4), 150.3 (C-2), 147.2 (C-1’), 139.7 (C-8a), 135.8 (C-7), 131.8 (C-2’, 6’), 128.1 (C-4’), 123.1 (C-5), 115.9 (C-6), 114.9 (C-8), 114.4 (C-4a), 110.7 (C-3’, 5’).

## Computational studies

### DFT Calculations

The DFT approach was used to compute the energies of FMOs and chemical reactivity descriptors based on FMOs for the synthesized 3-(arylamino)quinazoline-2,4(1*H*,3*H*)-diones derivatives **(3a − e)**. All DFT computations were done using the B3LYP/6-311G (2df, p) level of theory and the Gaussian 09W software; GuassView 6.0 was used to check files [24].

### Molecular Docking Studies

The crystallographic structures of MTH1 (4N1U) were retrieved from the RCSB database. All protein docking preparations were conducted using the Protein Preparation Wizard [30], where the protein was optimized, and missing residues were addressed. The synthesized derivatives were drawn using GuassView 6.0 and converted to.pdb files for ligand preparation. The OPLS_2005 force field was applied to prepare the ligand at a pH of 7.0 ± 2 [31]. Molecular docking simulations were performed using the Schrödinger software [32], specifically utilizing Glide with standard accuracy and flexible ligand sampling, which generated 26 Å grid boxes at each binding site and reported 10 poses per ligand. The 2D and 3D interactions were visualized using BIOVIA Discovery Studio [33].

### MM-GBSA Calculations

Prime MM-GBSA determines the binding free energy by evaluating the individual energies of the optimized apo-receptor, the free ligand, and the ligand-receptor complex. Additionally, it assesses the ligand strain energy by simulating the ligand in a solvent environment generated by the VSGB suite. The Prime energy visualizer facilitates the graphical representation of these energy calculations, providing a clear visualization of the energetic contributions [34].

### MD Simulation

An MD simulation was performed utilizing the Desmond software through the Schrödinger Maestro interface [35]. The findings were consistent with those obtained from the earlier docking procedure involving the complex. The simulation cell was characterized as orthorhombic and was populated with water molecules according to the SPC model. Additionally, the system was supplemented with sufficient ions to neutralize the overall charge of the complex. The duration of the simulation was 200 ns, operating under the NPT ensemble framework. Throughout the simulation, the number of atoms remained constant, with the pressure maintained at 1.01325 bar and the temperature at 300 K. The default thermostat employed was the Nose-Hoover chain method, with a 1.0 picosecond interval, whereas the Martyna-Tobias-Klein method served as the default barostat, set to a 2.0 picosecond interval. The MD simulation was evaluated using the Maestro simulation interaction diagram.

### Evaluation of Drug-Likeness and *In Silico* ADMET Prediction

The approach utilized for assessing the drug-likeness and pharmacokinetic profiles of the synthesized 3-(arylamino) quinazoline-2,4(1*H*,3*H*)-dione derivatives **(3a − e)** involved the SwissADME platform [36]. This methodology enabled the evaluation of several established drug-likeness criteria, including Lipinski’s Ro5, Veber’s Rule, Ghose’s Rule, and Egan’s Rule. These criteria were carefully applied to determine the drug-likeness profiles of the synthesized derivatives. Furthermore, *in silico* ADMET predictions were conducted using the pkCSM platform to analyze the ADMET of the compounds. This comprehensive strategy yielded valuable insights into the pharmacokinetic properties of the derivatives and their potential as promising drug candidates.

All data generated or analyzed during this study are included in this published article and its S1 File.

## Supporting information

S1 File The supporting information is available and provided copies of IR, ^1^H-NMR and ^13^C-NMR spectra for all products. (DOCX)

S2 File Synthesis of 3-(arylamino) quinazoline-2,4(1*H*,3*H*)-dione (3a) under Cu(NO₃)₂-mediated conditions. (DOCX)

S3 File Suggested mechanism for the generation of product (3a). (DOCX)
