# Supplementary material for: Synthesis of 3-(arylamino) quinazoline-2,4(1H,3H)-dione derivatives via TBHP/I2: Molecular docking, MD simulation, DFT, and pharmacological evaluation as MTH1 inhibitors
Source: PLoS One. 2025 Nov 13;20(11):e0335707. doi: 10.1371/journal.pone.0335707 (PMC12614599; doi:10.1371/journal.pone.0335707)

**Supporting Information**

**Synthesis of 3-(arylamino) quinazoline-2,4(1*H*,3*H*)-dione derivatives *via* TBHP/I_2_: Molecular docking, MD simulation, DFT, and pharmacological evaluation as MTH1 inhibitors**

Moeid Goudarzi Karim, Morteza Mehrdad*

, Davood Gheidari*, Nazanin Zahra Gheidari

*Department of Chemistry, Faculty of Science, University of Guilan, Rasht, Iran*

*Email*: [davoodgheidari@gmail.com](mailto:davoodgheidari@gmail.com); [davoodgheidari@phd.guilan.ac.ir](mailto:davoodgheidari@phd.guilan.ac.ir),

Tel:+981333690274

**The Table of Contents**

| **Title** | **Page** |
| --- | --- |
| Title, author’s name, address and table of contents | 1 |
| Experimental Section; General remarks | 2 |
| ^1^H and ^13^C NMR and IR and Mass spectrums of **3a** | 2-4 |
| ^1^H and ^13^C NMR and IR spectrums of **3b** | 5-6 |
| ^1^H and ^13^C NMR and IR spectrums of **3c** | 7-8 |
| ^1^H and ^13^C NMR and IR spectrums of **3d** | 8-9 |
| ^1^H and ^13^C NMR and IR spectrums of **3e** | 10-11 |

**Experimental Section**

**General remarks:**

Melting points were measured on an Electrothermal 9100 apparatus. Mass spectra were recorded with an Agilent 5975C VL MSD with Triple-Axis Detector operating at an ionization potential of 70 Ev. ^1^H and ^13^C NMR spectra were measured (DMSO) with a Bruker Bio Spin spectrometer at 300 and 75 MHz, respectively. IR spectra were recorded on a Bruker Tensor 27, ῡ in cm^-1^. Chemical shifts are reported in parts per million (*δ*) downfield from an internal tetramethylsilane reference. Coupling constants (*J* values) are reported in hertz (Hz), and spin multiplicities are indicated by the following symbols: s (singlet), d (doublet), t (triplet). All chemicals were purchased from Merck or Aldrich and were used without further purification.


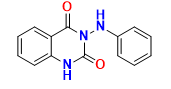

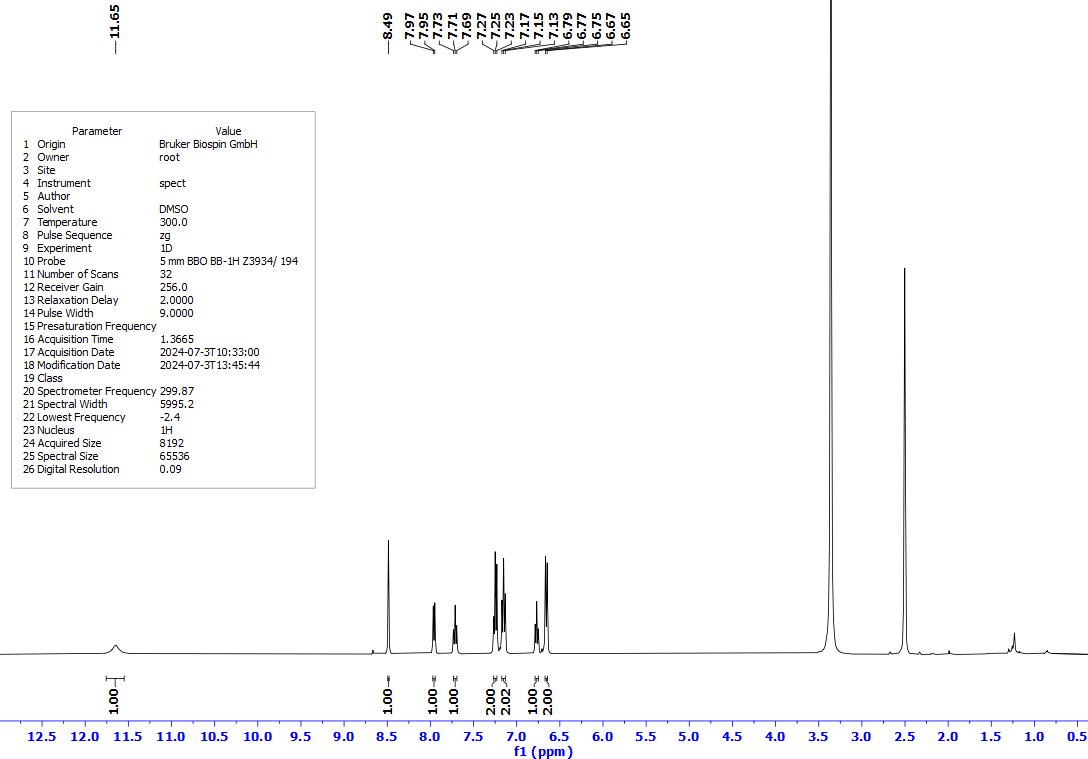


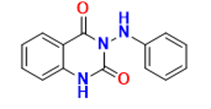



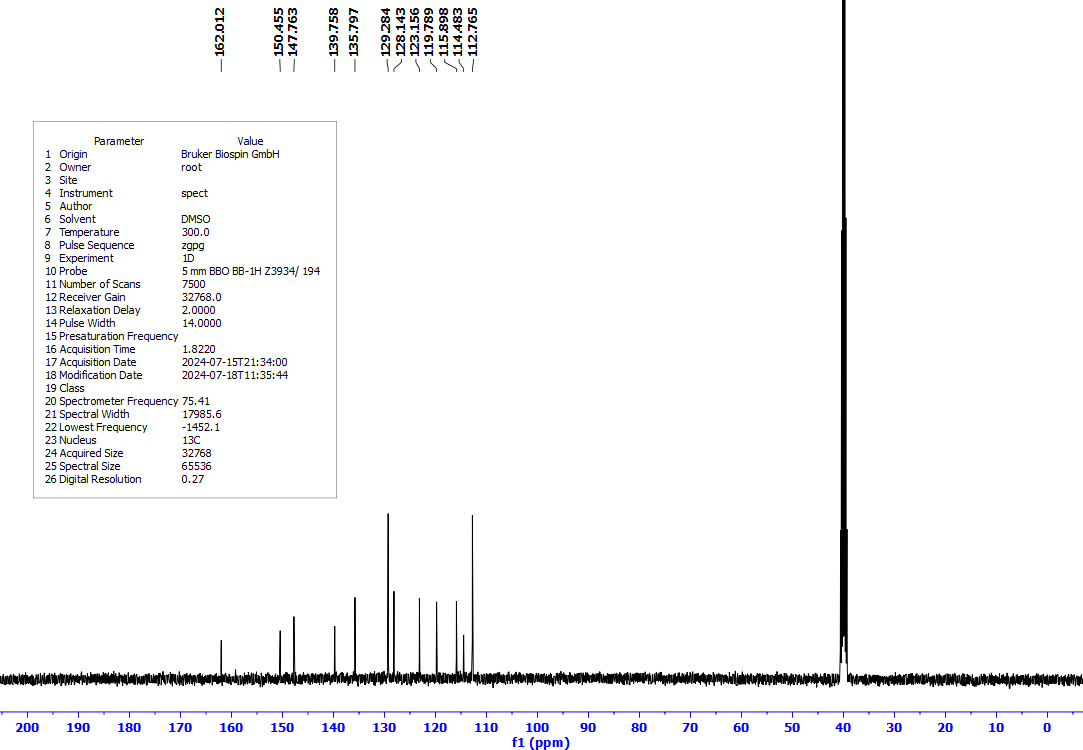


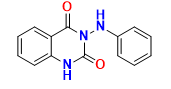

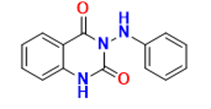


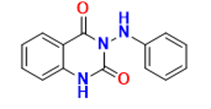


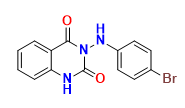

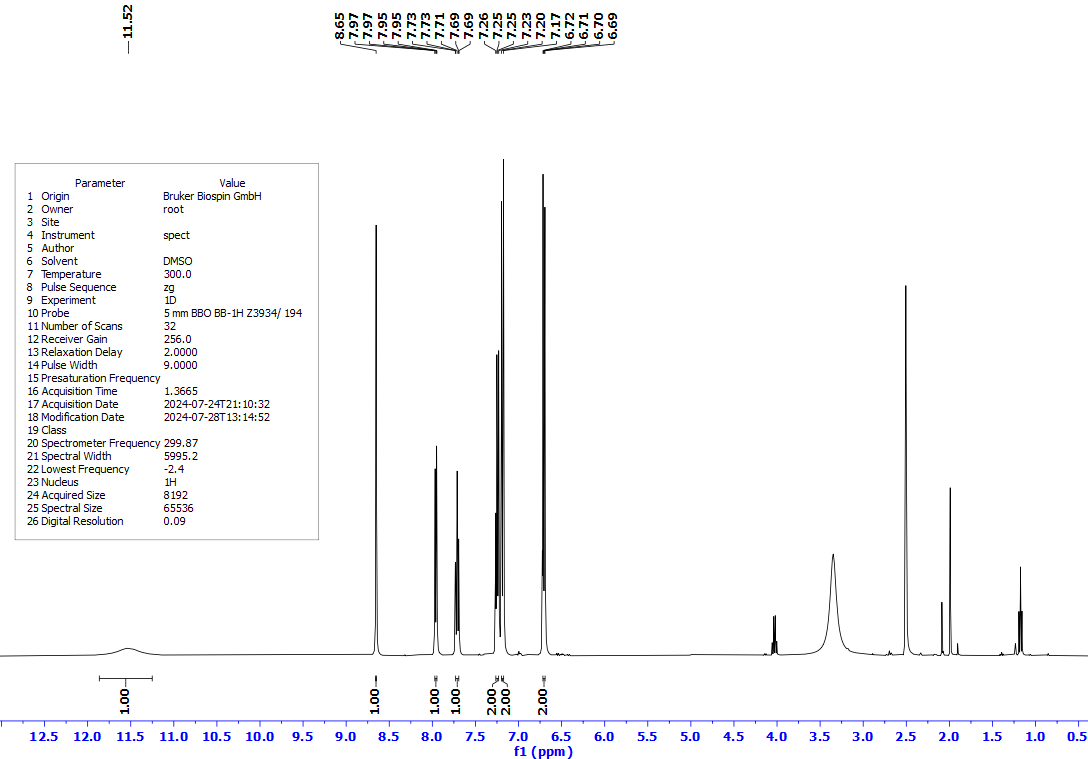


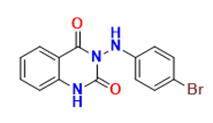

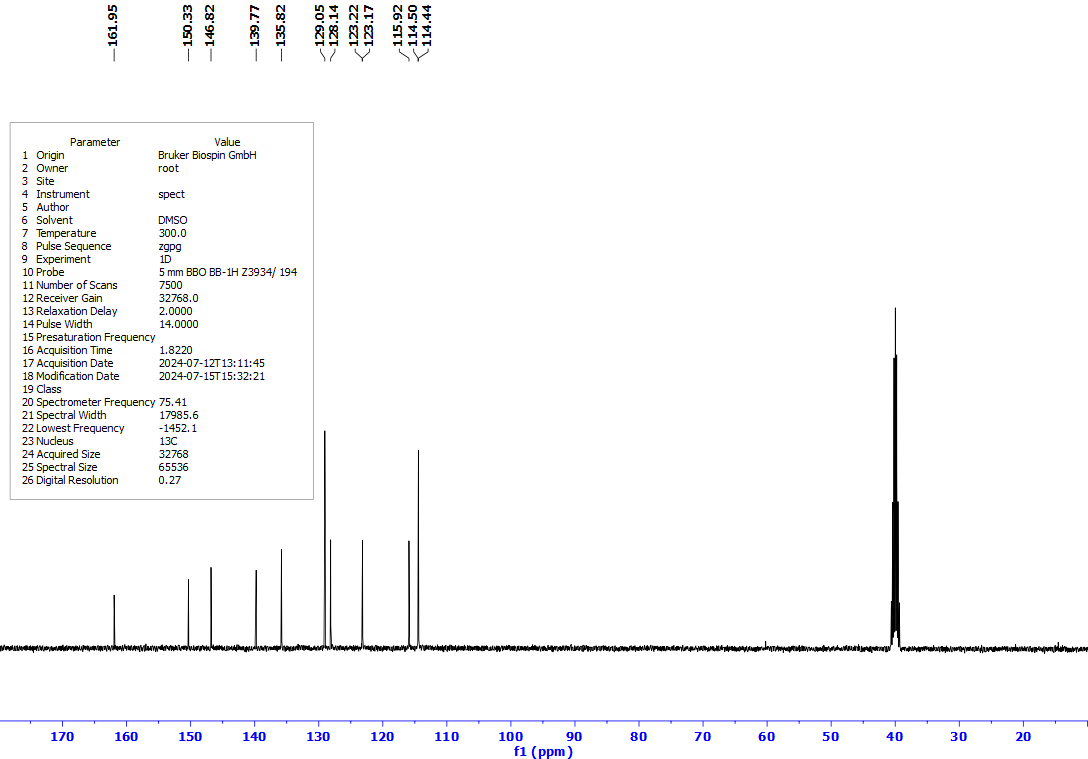


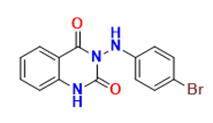




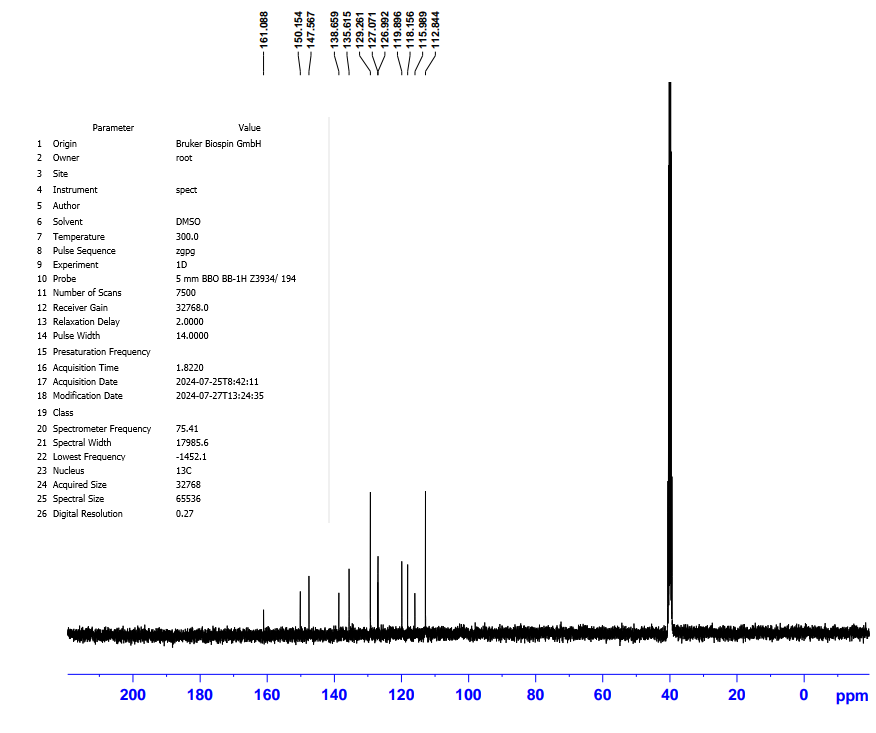

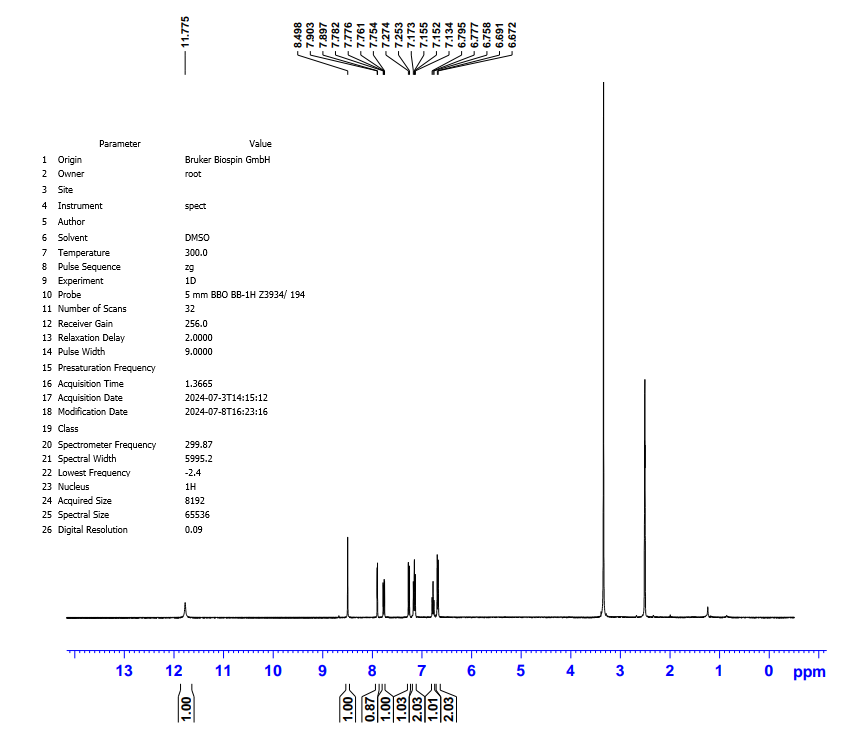

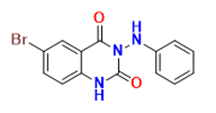

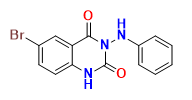


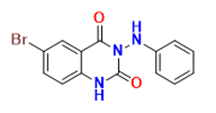




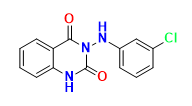

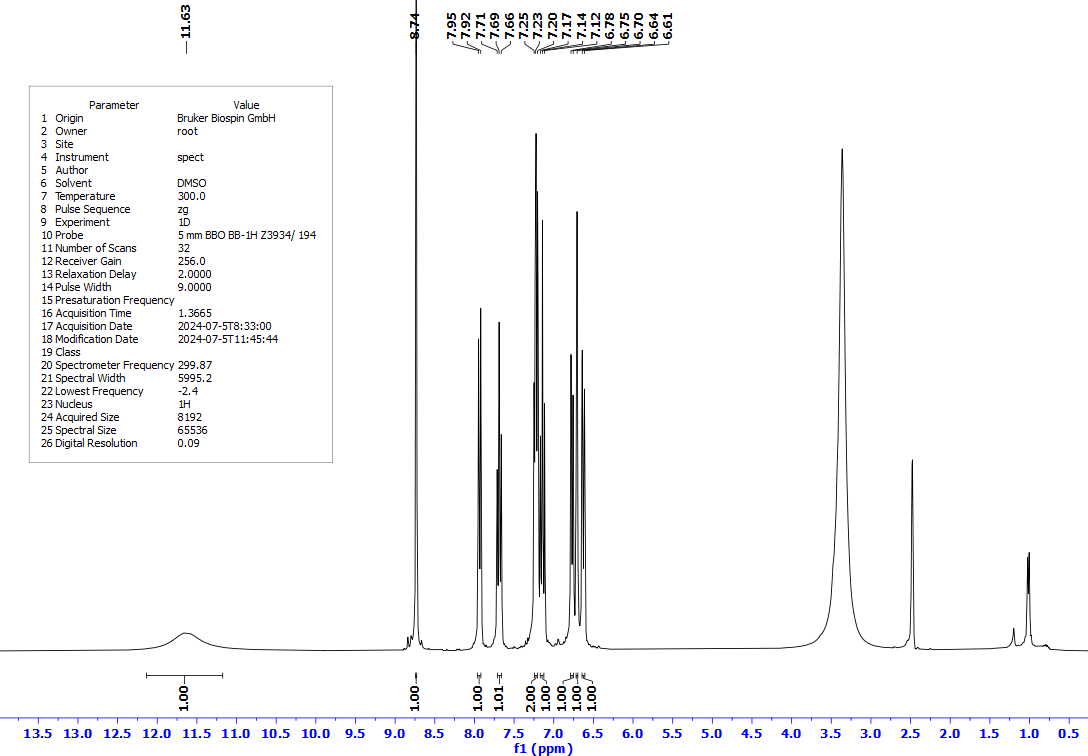


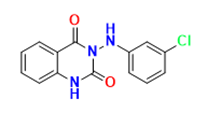


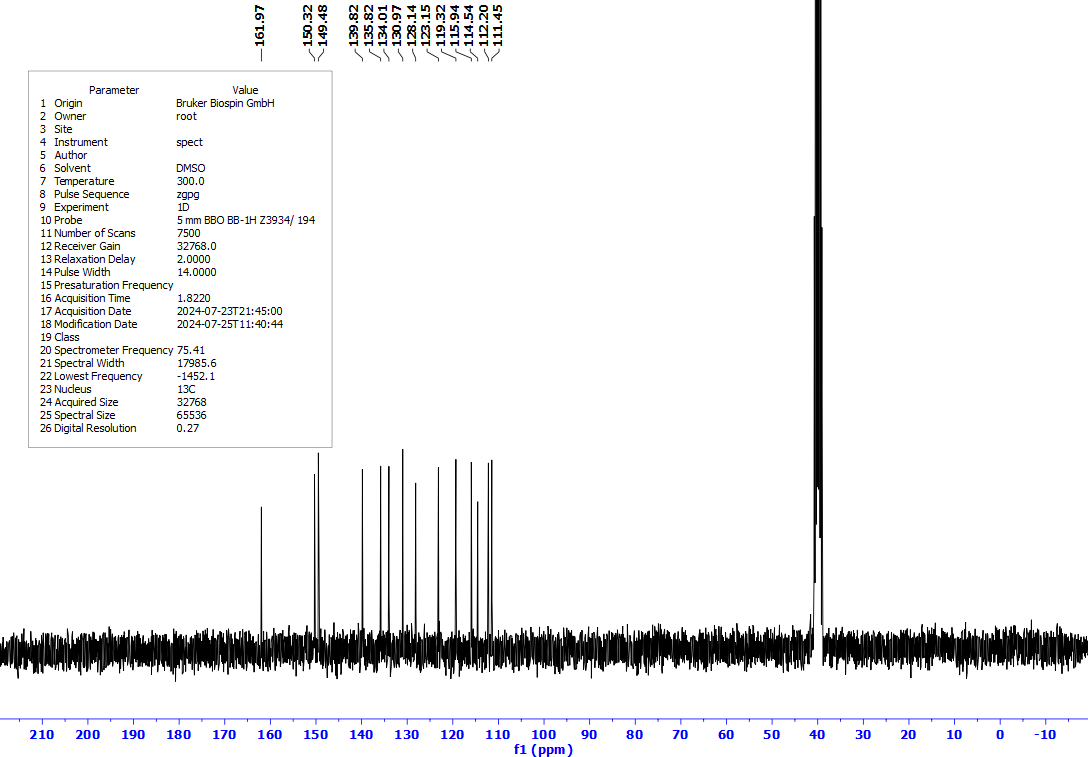


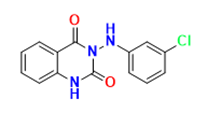




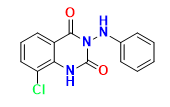


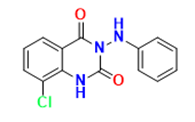

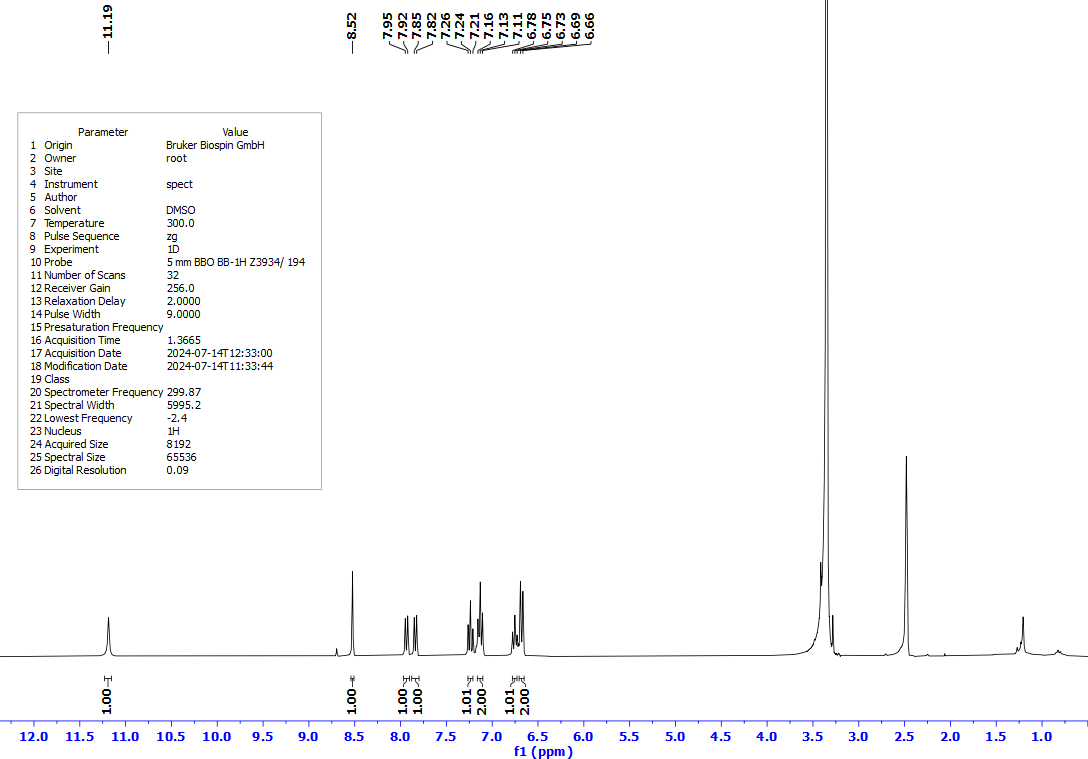


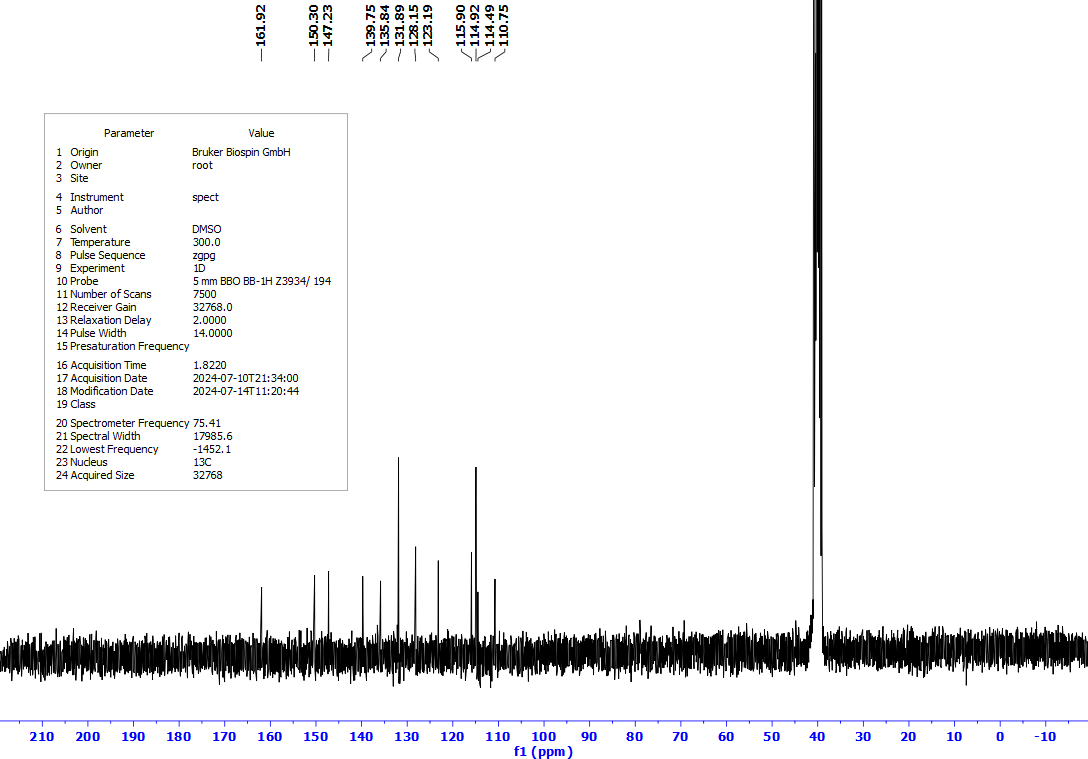


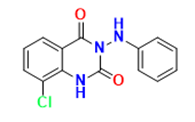

Supplement: S1 File — (DOCX) [file pone.0335707.s001.docx]
