# Supplementary figures and images for: Synthesis of 3-(arylamino) quinazoline-2,4(1H,3H)-dione derivatives via TBHP/I2: Molecular docking, MD simulation, DFT, and pharmacological evaluation as MTH1 inhibitors
Source: PLoS One. 2025 Nov 13;20(11):e0335707. doi: 10.1371/journal.pone.0335707 (PMC12614599; doi:10.1371/journal.pone.0335707)

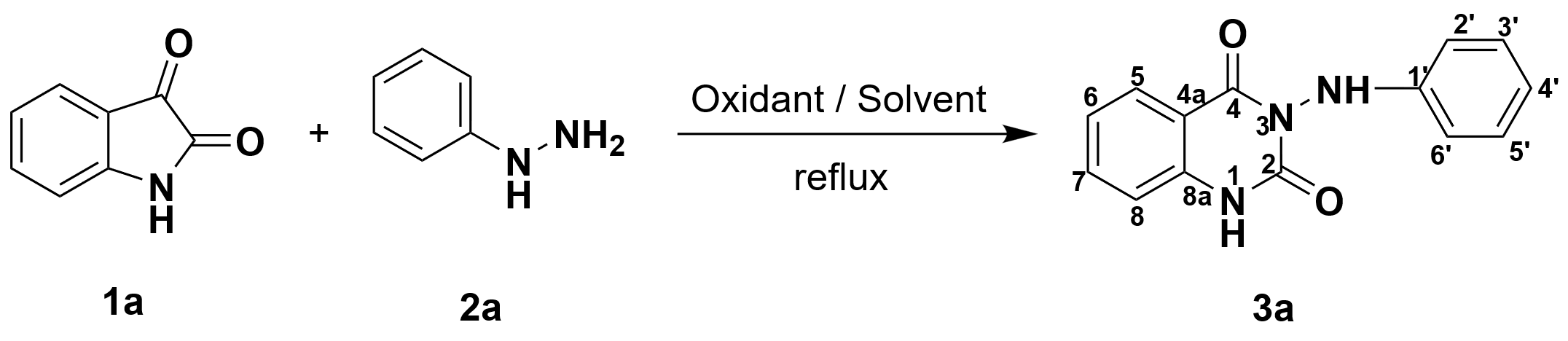

Supplement: S2 File — (DOCX) [file pone.0335707.s002.docx]
