# Supplementary material for: Synthesis of 3-(arylamino) quinazoline-2,4(1H,3H)-dione derivatives via TBHP/I2: Molecular docking, MD simulation, DFT, and pharmacological evaluation as MTH1 inhibitors
Source: PLoS One. 2025 Nov 13;20(11):e0335707. doi: 10.1371/journal.pone.0335707 (PMC12614599; doi:10.1371/journal.pone.0335707)
Supplement: Supplementary file 3 [file pone.0335707.s003.docx]

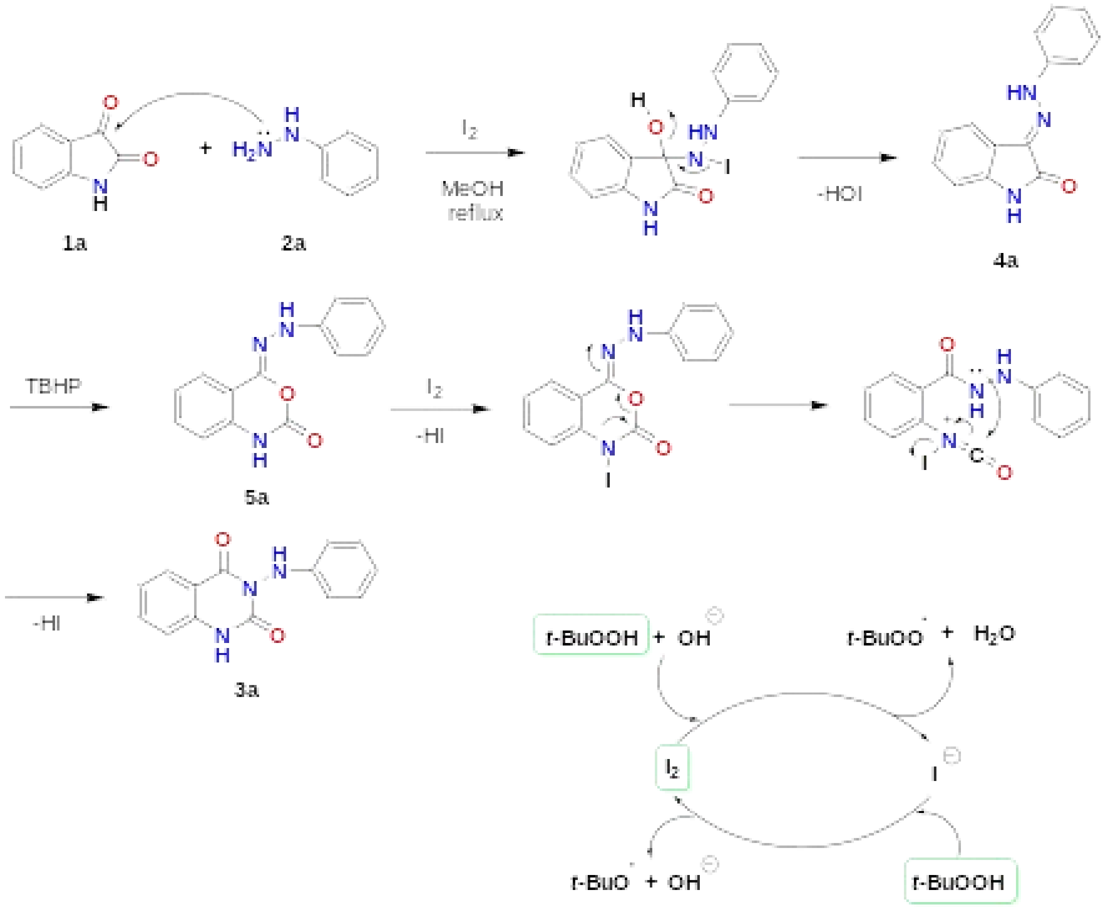
1a
2a
I2
MeOH
reflux
-HOI
4a
TBHP
5a
I2
-HI
-HI
3a
t-BuOOH + OH
t-BuOO + H2O
I2
I
t-BuO + OH
t-BuOOH
